# Penetration Coefficients of Commercial Nanolimes and a Liquid Mineral Precursor for Pore-Imitating Test Systems—Predictability of Infiltration Behavior

**DOI:** 10.3390/ma16062506

**Published:** 2023-03-21

**Authors:** Andra-Lisa Maria Hoyt, Marc Staiger, Marcel Schweinbeck, Helmut Cölfen

**Affiliations:** Department of Chemistry, Physical Chemistry, University of Konstanz, Universitätsstraße 10, 78457 Konstanz, Germany; andra-lisa.hoyt@uni-konstanz.de (A.-L.M.H.);

**Keywords:** complex coacervation, nanolime, calcite, liquid mineral precursor, porous material, infiltration, remineralization, Lucas–Washburn equation, capillarity

## Abstract

Nanolimes have been commercially available for over a decade as a remineralization agent for natural stone to combat deterioration. While they have been applied successfully and studied extensively, their penetration abilities in different materials have not yet been readily quantifiable in situ and in real time. Using two transparent pore-imitating test systems (acrylic glass (PMMA) and polydimethylsiloxane (PDMS)) and light microscopy, the penetration coefficients (*PC*s) of two nanolimes (CaLoSiL (CLS) and Nanorestore Plus (NRP)), as well as their solvents, were determined experimentally in square channels of about 100 µm diameter. Their *PC*s and those for a previously published glass–resin-based test system were also predicted based on measurable material parameters or literature values using the Lucas–Washburn equation. Additionally, a liquid mineral precursor (LMP) of calcium carbonate based on complex coacervation (CC) was investigated as an alternative to the solid particle dispersions of nanolime. In general, the dispersions behaved like their pure solvents. Overall, trends could be reasonably well predicted with both literature and experimentally determined properties using the Lucas–Washburn equation. In absolute terms, the prediction of observed infiltration behavior was satisfactory for alcohols and nanolimes but deviated substantially for water and the aqueous LMP. The commercially available PMMA chips and newly designed PDMS devices were mostly superior to the previously published glass–resin-based test system, except for the long-term monitoring of material deposition. Lastly, the transfer of results from these investigated systems to a different, nontransparent mineral, calcite, yielded similar *PC* values independently of the original data when used as the basis for the conversion (all *PC* types and all material/liquid combinations except aqueous solutions in PDMS devices). This knowledge can be used to improve the targeted design of tailor-made remineralization treatments for different application cases by guiding solvent choice, and to reduce destructive sampling by providing a micromodel for pretesting, if transferability to real stone samples proves demonstrable in the future.

## 1. Introduction

**Figure 1:**
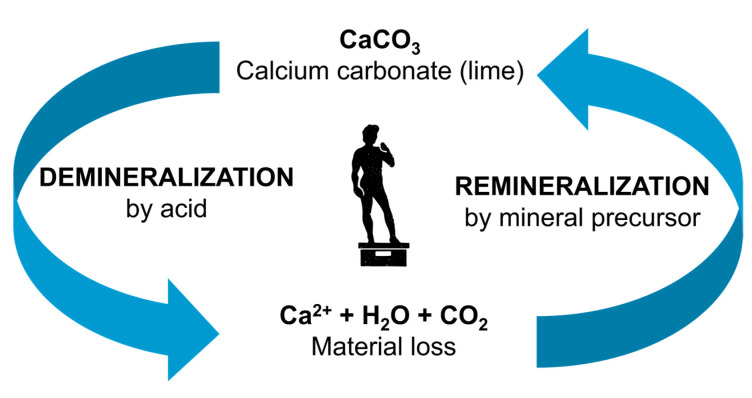
The cycle of calcium carbonate (lime) demineralization and remineralization, relevant to precious artifacts made of stone, e.g., the David statue (shown as silhouette “David” by Carpe Diem from Noun Project (CCBY3.0, https://creativecommons.org/licenses/by/3.0/, accessed on 6 March 2023) [13]).

Porous calcium-based minerals are and have always been of great importance and relevance in everyday life. They are, for example, an integral part of natural stone found not only in nature but also as part of humanity’s built and artistic cultural heritage. The porosity of these materials varies between rock specimens and is often heavily involved in their deterioration, where the first point of attack is the material’s surface, and its pores act as access points to the bulk material [1,2,3,4]. Accordingly, the porous structure must be considered when working with such materials. This makes an understanding of the infiltration behavior and transport processes in porous matter essential for the effective design and evaluation of new functional materials, the prevention of deterioration, and the optimization of restorative treatments. Cooperation between natural scientists and practitioners in the field is essential to leverage the newest scientific discoveries for the efficient treatment of porous materials in everyday life [5]. Examples include the remineralization of carious lesions in dental applications [2,6] and restorative treatments of cultural heritage [7]. While sealing teeth surfaces and pores have proven effective to slow down tooth decay [2,8], sealing stone surfaces with polymers was found to be largely detrimental to their conservation, which is why the development and optimization of new compatible inorganic treatments for stone consolidation are needed [5,9]. A promising material class for this purpose is nanolimes (NL), i.e., nano-scaled calcium hydroxide particle dispersions in alcohol [10,11,12]. The cycle of calcium carbonate mineralization is shown in Figure 1.

While there are many successful case studies on the use of NLs to treat damaged wall paintings or stone [14,15,16,17,18,19,20] and saving cultural heritage is a race against time [5,21,22], it is currently not possible to reliably predict the infiltration behavior of complex liquids in different substrates. All possible new combinations of substrate and restoration treatment must be explored almost completely via trial and error on actual (often precious and (semi-)destructively taken) samples or directly on the object [23], aided by previous experience and educated guesswork. Additionally, due to the nontransparency of stone samples [24], the infiltration behavior of liquids into their pores could so far only be monitored indirectly, time-delayed, resource-intensely, or destructively [14,15,20,23,25,26,27]. Furthermore, particle dispersions used for infiltration treatments must be tailored to pore size distributions of the substrate to limit the clogging of pores and surface coating formation [28].

A possible solution to the described methodical gap was presented by us in 2017, in the form of an artificial pore-imitating microcomb test system (MCTS) [29]. It constituted a new example of so-called micromodels which have been utilized extensively in the literature [24,30] and enable the direct visualization of pore-scale processes relevant to different porous media [31,32]. Their ability to directly monitor infiltration, drying, and (re)crystallization processes in confined geometries and real time using light microscopy was demonstrated by Gruber and Wolf et al. (2017), using an aqueous calcium phosphate crystallization solution [29]. The imitation of the target material was achieved by tuning the wettability of the device to match hydroxyapatite for dental applications and two calcite-containing rock species for stone conservation. This was achieved by coating the MCTS with thin layers of chromium and gold combined with a subsequent application of mixed thiol–gold self-assembled monolayers (SAMs) to create more hydrophilic or hydrophobic surfaces [29,33,34,35]. This system was later used to study the infiltration, drying, and crystallization process of commercial NLs as well as calcium-based complex coacervates (CCs) as a potential novel remineralization solution in pores that were also modified to imitate rock [35]. CCs of calcium belong to the so-called liquid mineral precursors (LMPs), a class of materials that is part of the calcium carbonate polyamorphism [36] and can be influenced by additives [37,38]. 

**Figure 2:**
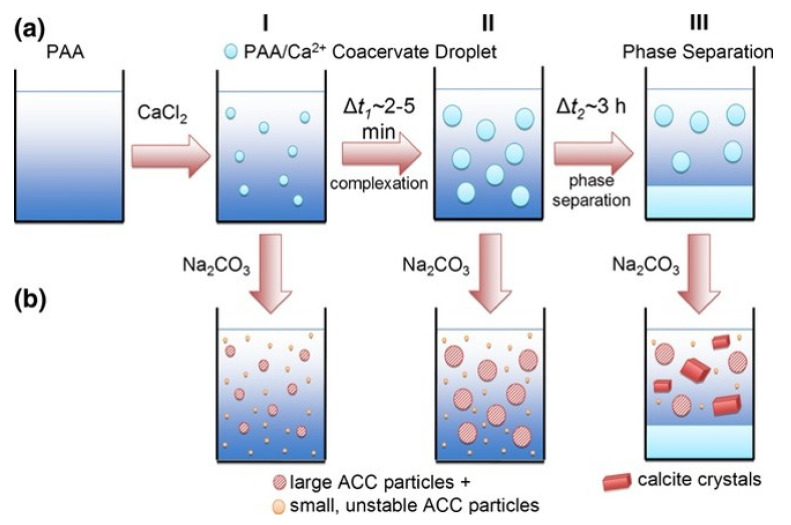
Scheme of the complex coacervation system: (**a**) the PAA/Ca^2+^-system shows time-dependent growth of the coacervate droplets; and (**b**) Addition of the Na_2_CO_3_ solution to the PAA/Ca^2+^ after different growth times (I, II, and III). Reprinted with permission from Maas et al. [40].

The general principle of coacervation was postulated about a century ago [39] but only applied as complex coacervation to the system of calcium and poly(acrylic acid) (PAA) in the last decade [40]. The coacervation process of polyelectrolytes in general [41] and PAA specifically [42] have been extensively investigated in recent years, showing promise for tailoring the properties of the produced materials. PAA is also already widely used in conservation efforts, e.g., as a contaminant capture agent [43] as well as macroscopic glue. CCs form via spontaneous liquid–liquid phase separation between two oppositely charged components, usually a smaller ion and a larger poly-charged molecule resulting in highly concentrated dense liquid droplets in a less dense surrounding aqueous phase (see Figure 2) [40]. They have been investigated for applications in drug delivery, tooth remineralization, fragrance encapsulation, and the deposition of material within the aforementioned MCTS [6,35,44,45] and are also being discussed as important species in the emergence of life on earth [46]. Especially, their liquid nature makes them very promising for the infiltration of porous substrates in need of remineralization, making adjustments to substrate pore size distribution obsolete.

While the MCTS constituted a significant step forward in the efforts to visualize and understand the behavior of liquids in porous materials for stone conservation, it exhibited several issues [29,35]. The most significant of these was the connectivity of the single device parts: not all channels were successfully sealed off tightly. Higher pressures result in the breakage of the brittle materials, glass, and photoresin. Small gaps between the parts exhibited significantly higher capillary suction than the designated pores themselves, leading to fluid and material deposition in undesired regions. Additionally, the use of closed pores results in air being trapped inside the channels, hindering the infiltration of liquids into the pores [47], and the reproducibility of hard photolithography was poor. Furthermore, the infiltration process itself and infiltration velocities were not monitored continuously but only at certain time points [29].

This publication describes the fabrication of an alternate micromodel made from polydimethylsilane (PDMS) with covalent functionalization and bonding based on soft photolithography for microfluidics [24,48,49]. Fabrication by molding enables the production of modified pore geometries to combat air entrapment. The surfaces were functionalized by plasma activation and subsequent poly(ethylene glycol) (PEG)–silane treatment [50] to imitate the wettability of calcite as a model system due to its relevance for cultural heritage as a component of calcitic stones. Commercially available poly(methyl methacrylate) (PMMA) microfluidic chips were also investigated. The devices were used to characterize the infiltration behavior of water, ethanol, isopropanol, and four different commercially available nanolime materials (Nanorestore (NRP) and CaLoSiL (CLS)) based on light microscopy infiltration experiments. Additionally, a liquid calcium mineral precursor formulation [35] based on the complex coacervation of calcium with poly(acrylic acid) [40,44] was investigated as a possible new consolidant for stone. Penetration coefficients (*PC*s) of all test system/liquid combinations were determined [47,51,52] based on the original and unmodified Lucas–Washburn (LW) equation [53,54]. In addition, *PC*s were also calculated for the previously published MCTS and compared to results from both of the other micromodels investigated here. Lastly, the determined *PC*s are tested for convertibility into different liquid/device combinations, namely water/calcite as a highly relevant reference system for the restoration of carbonatic stone, with the goal of demonstrating the predictability of infiltration behavior from experimental and theoretical data for arbitrary target systems. This would enable the targeted design of treatment solutions for individual case studies on a scientific and experimentally measurable basis.

## 2. Materials and Methods

### 2.1. Materials

Water refers to double-deionized water purified using a *Milli-Q Direct 8* machine from Merck Millipore (Darmstadt, Germany) with a resistivity of 18.2 MΩ cm. Si wafers of about five cm in diameter were purchased from Siegert Wafer GmbH (Aachen, Germany). Lithography masks and wafers were rinsed with analytical-grade isopropanol for cleaning. All of the following products were used directly as purchased without further purification or drying steps: Photo resin SU8-3050 and developer mrDev-600 purchased from Micro Resist Technology GmbH (Berlin, Germany). Nanorestore Plus Testkit (100 mL in ethanol, 5 g/L; 100 mL in isopropanol, 5 g/L) obtained from CSGI Headquarters, University of Florence (Florence, Italy). CaLoSiL Samplebox 1 (Fresco; 3 × 0.1 L of CaLoSiL^®^ E5, IP5, and E25 gray; 20 mL of pasty) containing the used CaLoSiL E5 and IP5 manufactured by IBZ—Salzchemie GmbH & Co. KG (Halsbrücke, Germany). PDMS components (Sylgard™ 184 Silicone Elastomer Kit) purchased from Dow (Wiesbaden, Germany). (Heptafluoropropyl)trimethylsilane for template hydrophobization as well as thiols for gold surface modifications (mercapto-1-undecanol), sodium hydroxide, hydrochloric acid solutions (1 N and 0.1 N), and poly(acrylic acid) sodium salt solution (PAANa, 15 kDa, 35 weight% in water) purchased from Sigma-Aldrich via Merck KGaA (Darmstadt, Germany). 1-Dodecanethiol (DDT, ≥98%) originated from Acros Organics (Geel, Belgium). Three-[Methoxy(polyethyleneoxy)propyl]trimethoxysilane (90%, 6–9 PE units) was supplied by abcr GmbH (Karlsruhe, Germany). Absolute ethanol, reaction-grade isopropanol, and acetone supplied by VWR (Darmstadt, Germany). Calcium chloride dihydrate (CCDH) was purchased from Carl Roth GmbH+ Co. KG (Karlsruhe, Germany). Calcite specimen from Schmuckstein-Shop 24 (Idar-Oberstein, Germany). 

### 2.2. Device Fabrications

#### 2.2.1. Soft Lithography Route

**Figure 3:**
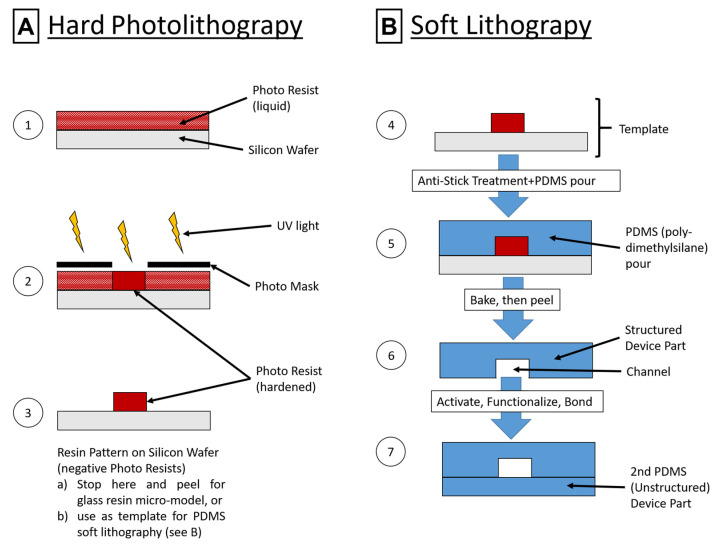
Schematic illustrations of the hard photolithography process (**A**), as used for the glass–resin microcomb test system, consisting of coating a silicon wafer with photoresin and pre-baking (1), partial illumination with a mask for structured crosslinking (2), and development of the resin on the wafer to yield the illuminated structure. (**B**) shows the following process of soft lithography, as used for the PDMS micromodel, where the result from hard photolithography (3) is used as a template (4) for PDMS imprint manufacture after and additional anti-stick treatment. PDMS is freshly mixed and poured on to the template (5), then baked before peeling (6), activation, functionalization and bonding, yielding a microchannel in PDMS (7). Based on source [57] with permission.

**Figure 4:**
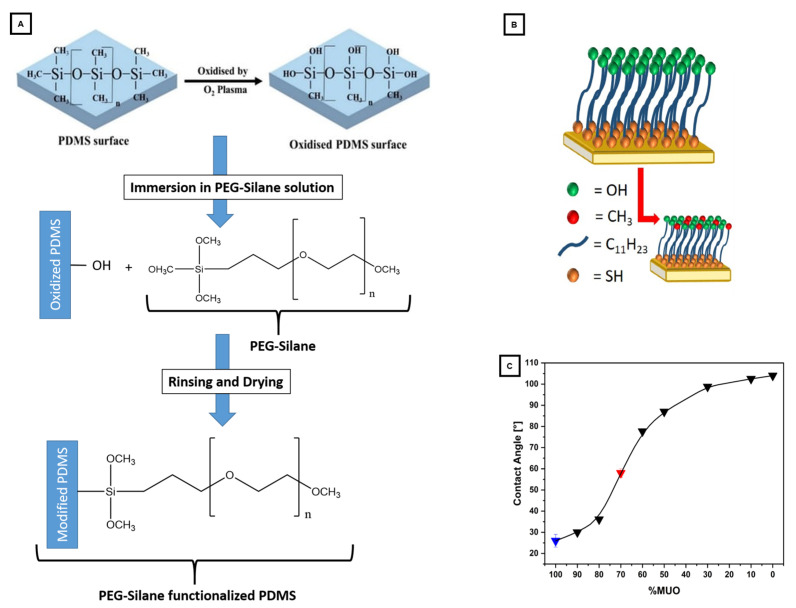
(**A**) Chemical representations of the activation and functionalization of PDMS surfaces using oxygen plasma and polyethyleneglycol(PEG)-silanes. The top part was reused from the source [61]. The bottom part was based on the source [50] and adapted. (**B**) Sketch of a thiol–gold interaction-based self-assembled monolayer on a gold-coated surface made from a mixture of hydroxyl- and alkyl-terminated alkanethiols, modified from source [29]. (**C**) The resulting contact angles (CAs) for different SAMs from the most hydrophilic (low CA and high %MUO) to the most hydrophobic (high CA and low %MUO), based on source [29]. The blue triangle signifies the mixture used for sandstone imitation, and the red triangle the mixture used for marble/calcite/enamel imitation. The reuse and reprint of all images and image parts are licensed under CCBY4.0 (https://creativecommons.org/licenses/by/4.0/, accessed on 6 March 2023) from cited sources.

**Figure 5:**
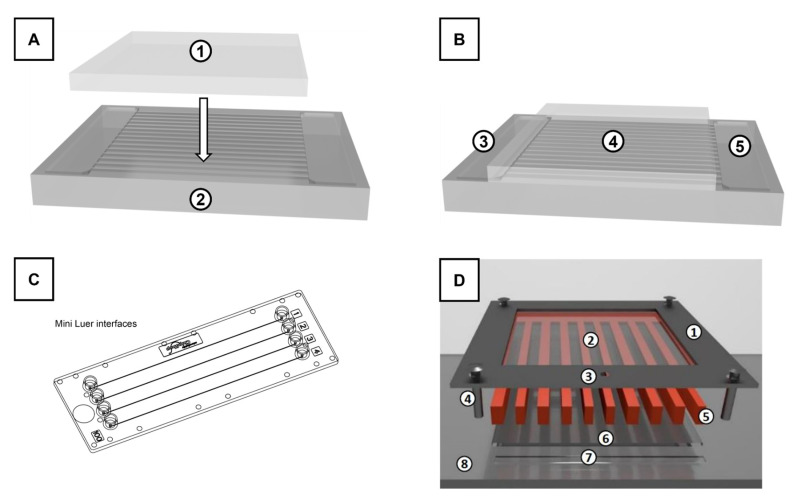
Illustrations of the three discussed infiltration devices. PDMS-based soft lithography (top), showing the unstructured rectangular PDMS piece as a lid (1) and structured PDMS piece (2) in disassembled (**A**) and assembled state (**B**) with the reservoir for liquid application (3), closed channels formed between the base piece and the lid (4), and the outlet reservoir for air escape and liquid accumulation (5). PMMA-based commercial chips (**C**) with four inlets, channels, and outlets. SU8/Glass system ((**D**), [29]) with metal frame for pressure application from the top (1), top glass platelet (2), inlet for liquid (3), screws securing the metal frame to the base plate (4), microcomb made from structured photo resin (5), bottom glass platelet (6), infiltration edge (7), acrylic base plate (8). Copyright for image (**C**) remains with *microfluidic ChipShop GmbH*, reprinted with permission. Image (**D**) is taken from source [29] by Gruber/Wolf et al. under a CCBY4.0 license (https://creativecommons.org/licenses/by/4.0/, accessed on 6 March 2023).

Procedures for the soft lithographic route were based on previous works for hard photolithography [29,35] and soft lithography [55,56], adapted as necessary. The general processes are illustrated in Figure 3. Master templates: A 5 cm Si wafer was spin-coated with about 2 mL of SU8-3050 photo resin for 5 s at 500 rpm (acceleration: 100 rpm/s) and for 30 s at 1950 rpm (acceleration: 300 rpm/s). Prebake was performed at 90 °C for 45 min, followed by 4 × 10 s light exposure waiting for 10 s between illuminations using soft contact mode and a mask (Supplementary Materials, Figure S1). Postbake consisted of 1 min at 65 °C and 15 min at 95 °C. Films were developed for 3 × 8 min in a fresh developer solution and rinsed with isopropanol. After drying in a nitrogen stream, the completed master template (MT) was characterized via profilometry (Supplementary Materials, Figure S2, Tables S1 and S2). A blank Si wafer was used as a template for the nonstructured lid. Both templates were treated with 5 µL of (heptafluoropropyl)trimethylsilane in an evacuated desiccator for at least 2 h, repeated every five imprints. The templates can be cleaned in isopropanol and reused indefinitely until mechanical damages occur. Printing: PDMS was prepared according to the manufacturer’s recommendations (10:1 = monomer:curing agent). About 2.8 g of PDMS was poured for a structured print and 2.4 g for a lid. Samples were placed in an oven at 65 °C for at least 2 h. Afterward, the PDMS was cut and peeled using isopropanol as a separating agent. Structured PDMS was cut to 3.5 × 2.5 cm and lids to 2 × 2.5 cm, then rinsed and stored with the formerly Si-facing side upward. Functionalization and assembly: The functionalization procedures were inspired by reference [50] and modified (see Figure 4A). The upward-facing side was activated using a reactive ion etch process (42 µbar 100% oxygen plasma, 30 W, and 90 s) and then immediately submerged in 20 mL of a 0.1 mol/L 3-[Methoxy(polyethyleneoxy)propyl]-trimethoxysilane solution in ethanol for 20 min under slight agitation. The device parts were placed in fresh ethanol solution twice for washing (30 min and 10 min). After rinsing with ethanol, they were dried in a 40 °C vacuum oven for 2 h. Bonding was achieved by a second oxygen plasma treatment at about 100 W for 27 s, with immediate assembly and subsequent heat treatment for 15 min at 100 °C on a hotplate. The resulting devices (*PDMS* for modified PDMS) were investigated using light microscopy (Supplementary Materials, Figure S2, Tables S3 and S4) and are shown schematically in Figure 5A,B.

#### 2.2.2. Poly(methyl methacrylate) (PMMA) Microfluidics

For the commercial poly(methyl methacrylate) (PMMA) system, a straight channel chip with four parallel channels of type Fluidic 144 (product code 10000193) was purchased from microfluidic ChipShop (Stockholmer Str. 20, 07747 Jena, Germany) and used as supplied. Specifications for channel width and depth were 100 µm, with a length of 58.5 mm. The lid thickness was given as 175 µm. The material of the chip was poly(methyl methacrylate) (PMMA), also known as acrylic glass or plexiglass. See Figure 5C for a scheme.

#### 2.2.3. Glass–Resin Microcomb Test System

The investigations to simulate infiltrations into the previously published glass–resin microcomb test system [29,35] (see Figure 5D) were executed using precleaned coverslips (15 mm edge length and 0.3 mm thickness) coated in 5 nm of chromium and 20 nm of gold. Self-assembled monolayers (SAMs) were achieved by treatment with a mixture of MUO and DDT in ethanol for about 16 h. The mixed SAM is illustrated schematically in Figure 4B along with the resulting contact angles according to the authors of reference [29] in Figure 4C [29]. Due to varying thiol quality, a higher MUO to DDT volume ratio of 8:2 was used for this current study instead of 7:3 to reach a similar contact angle (CA).

### 2.3. Consolidants

#### 2.3.1. Nanolimes

Nanorestore Plus (NRP) consists of Ca(OH)_2_ (calcium hydroxide) nanoparticles dispersed in alcohol (E: ethanol; iP: isopropanol) at concentrations of 5 g/L. For NRPiP, the nanoparticles were hexagonal portlandite platelets between 20 and 200 nm [58]. CaLoSiL (CLS) E5 and IP5 also consist of 5 g/L Ca(OH)_2_ nanoparticles in alcohol. For CaLoSiL in general, sizes lie between 50 and 250 nm [59,60]. The mean values for the batches used were 123 nm for CLSiP 5 and 125 nm for CLS-E, according to accompanying documentation.

#### 2.3.2. Complex Coacervate Formulation

The complex coacervate (CC) recipe was based on previous work and the principle of complex coacervation of calcium first described by Maas et al. [35,40,44]; See Figure 2 for an illustration. 0.2 M calcium chloride dihydrate (CCD) and 0.5 mg/mL aqueous poly(acrylic acid) sodium salt (PAANa) solutions were prepared and set to a pH value of about 8 using small amounts of sodium hydroxide and hydrochloric acid solutions. For coacervate generation, 1 mL of CCD solution was added to 1 mL of PAANa solution in a snap-on cap vial, shaken vigorously, and then immediately used. A slight turbidity increase signifies the presence of CCs in the form of liquid droplets in an aqueous phase. 

### 2.4. Infiltration Experiments

Infiltration experiments were conducted into modified PDMS (*) and PMMA devices with water, ethanol, and isopropanol, as well as Nanorestore Plus (NRP) and CaLoSiL (CLS) in ethanol or isopropanol, and the complex coacervate (CC) formulation. Due to differences in device geometries, their infiltration procedure also differs. *PDMS* devices consisted of 17 channels, 14 of which could be monitored simultaneously with a light microscope. All channels were infiltrated at the same time from their shared reservoir. Infiltrations were performed using 20 µL of alcohols or their dispersions. For aqueous CCs and water, 100 µL were applied due to higher surface tension preventing the liquid from spreading to all channels at lower volumes. PMMA device infiltrations were performed channel by channel with 18 µL of liquid per infiltration. One chip (four channels) was used per liquid. Chips used for water or CC infiltration could be reused after rinsing with water and drying. Since alcohols destroy PMMA [24], the chips used for these infiltration experiments were not reused but discarded after the experiments. 

### 2.5. Determination of the Penetration Coefficient

To determine the penetration coefficient of different material/liquid combinations, the capillarity principles described by the Lucas–Washburn (LW) equation are essential [53,54]:

(1) $$d\;\left(t\right)=\sqrt{\frac{\sigma *\cos\theta }{2\eta }\;\cdot \;r\;\cdot \;t}$$

with traveled distance *d* of the meniscus at time *t*, surface tension *σ*, viscosity *η*, the mean pore radius *r,* and contact angle (CA) *θ* between liquid and substrate. This theory assumes regular round capillaries in a horizontal position, with negligible impact of gravity, and in contact with an unlimited liquid reservoir.

An important part of this equation describes the properties of the liquid and its interaction with the solid material of the capillary independently of capillary radius *r* and time *t.* It is the so-called penetration coefficient *PC* [47,51,52]:

(2) $$PC=\frac{\sigma *\cos\theta }{2\eta }$$

There have been many modifications and refinements of the LW equation, as summarized by Cai et al. for the description of flow in porous systems [62]. We are interested in the question of whether the unmodified LW equations could be sufficient for practical conservators with a limited mathematical background and technical equipment.

### 2.6. Predictions of Penetration Coefficients

In order to later predict the infiltration behavior of liquids in calcite materials (such as marbles or limestones), the most relevant question is how much a given liquid infiltrates into a certain material in a given time, described by the *PC* of calcite with this liquid. Therefore, the *PC*s determined before according to Equation (2) need to be converted as follows:

(3) $$cPC_{1}\left(2\right)=PC_{2}*\;\frac{\sigma_{1}*\cos\theta_{1}*\eta_{2}}{\sigma_{2}*\cos\theta_{2}*\;\eta_{1}}$$

where the subscripts denote one specific combination of material and liquid. Converted *PC*s are denoted as *cPC;* the material/liquid combination used as the basis for conversion is given as input in brackets and the resulting material/liquid it was converted into as subscript, e.g., c*PC*_PMMA/water_ (*PDMS*/EtOH) is the converted *PC* of PMMA in water based on the *PC* of *PDMS* with ethanol. For the same liquids or the same materials, the denotation of the duplicate liquid or material can be omitted if clear from the context. When the liquid is the same for both combinations, then the equation can be simplified to:

(4) $$cPC_{1}\left(2\right)=PC_{2}*\;\frac{\cos\theta_{1}}{\cos\theta_{2}}$$

This way we can theoretically make predictions for the infiltrations of liquids into nontransparent materials, such as stone, based on experimental or calculated infiltration parameters, such as the *PC*, to enable the targeted design of treatments.

## 3. Results and Discussion

### 3.1. Device Fabrication

For this study, a previously published microcomb test system (MCTS) [29,35] was reviewed and compared to an alternative micromodel based on soft lithography microfluidics as well as a commercially available microfluidic chip. A schematic of single (A) and assembled (B) polydimethylsiloxane (PDMS) microfluidic device parts is given in Figure 5. In Figure 5C, the applied commercial poly(methyl methacrylate) (PMMA) chip is illustrated. The previously published microcomb test system (MCTS) based on photoresin microcombs and a holding apparatus is depicted in Figure 5D. 

#### 3.1.1. Soft Photolithography Route

The production of PDMS-based devices encompasses three main steps: the manufacture of the master template (MT), the printing of the PDMS device part, and lastly its functionalization and assembly.

Manufacture of the MT and PDMS imprints: Once a suitable MT is produced, imprinting PDMS parts is a very cheap and reproducible step when the hydrophobization of the MT is regularly renewed and mechanical damage is avoided. All PDMS imprints were manufactured using the same MT with 17 channels and two reservoirs. Channel dimensions in PDMS were on average 92 ± 1 µm by 112 ± 3 µm with a cross-sectional area of 10,363 ± 257 µm^2^ (Supplementary Materials, Figure S2, Tables S3 and S4). Their shape can be described as rectangular with irregularities where protruding PDMS connects to the PDMS base. The flexible nature of PDMS compensates for small shapes or surface irregularities and provides a good seal. The uniformity of both the template and the imprint was demonstrated to be very good with variations between 2–5% along the channels. The investigation of the actual imprints used as part of the device by light microscopy can be assumed to be the more accurate representation of channel geometries compared to the trapezoidal appearance in the MT. The difference can be explained by the different techniques employed: contact profilometers, as used for the template, have a finite tip dimension (here: 2 µm). This leads to a distortion of the detected profiles, especially if they consist of many small protrusions, where the tip cannot accurately map the valley between the hills. This explains the overly wide basal width of the template (252 µm), which is not as pronounced in the PDMS imprint (141 µm). 

**Figure 6:**
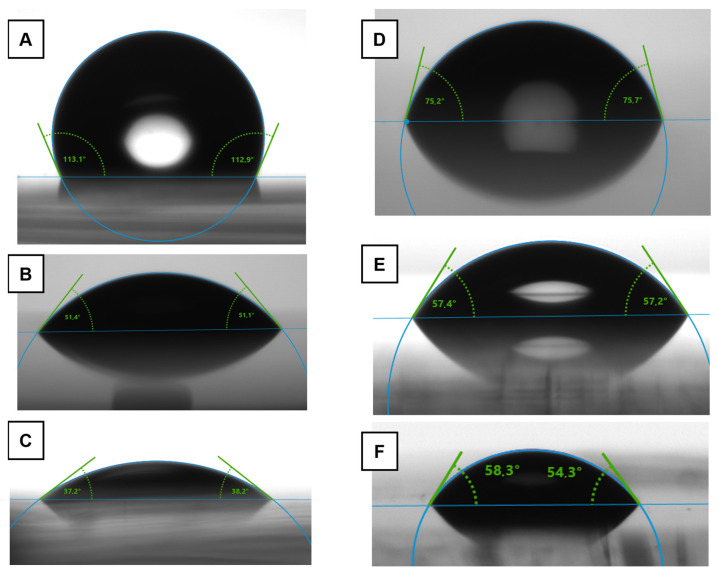
Contact angles of water measured on (**A**) native PDMS, (**B**) PEG-silane-functionalized PDMS, (**C**) functionalized and bonded PDMS; as well as (**D**) native PMMA, (**E**) SAM on the gold surface with an 8:2 volume ratio of MUO:DDT, as present in the modified SU8 system, and (**F**) Calcite.

Functionalization: To hydrophilize the naturally hydrophobic PDMS, a process described by Demming et al. [50] was employed, using plasma oxidation to activate the PDMS surface, which then can be directly bonded to another activated PDMS surface or glass. For hydrophilization, PEG-silane chains were attached to the activated PDMS surface, which then presents a new, more hydrophilic surface after rinsing and drying (see Figure 4A). The effectiveness of the hydrophilization was determined by measuring the contact angles (CAs) and free surface energies (*SFEs*) of the PDMS before (native) and after treatment (*) (Figure 6A–C,). The procedure used for this study resulted in a reproducible reduction in CAs from 113° for native PDMS to about 51° (compared to the literature: the reduction from values around 115° to values as low as 10° [50]). The very low values in the literature might be due to insufficient removal of excess PEG-silane chemical from the PDMS surface since a constant CA of about 50° is reached after a week of storage [50], which agrees quite well with the measurements presented here. The *SFE* of PDMS increased about fourfold to 62 mN/m after hydrophilization, with a distinctly higher polar fraction of 31% compared to 3% before. 

The long-term stability of this functionalization is limited and must be monitored to determine which CAs can be assumed during infiltration. This limitation is due to “hydrophobic recovery caused by the migration of uncured PDMS oligomers from the bulk to the surface and the rearrangement of highly mobile polymer chains featuring Si-OH bonds toward the bulk at room temperature” [63]. Directly after functionalization, the difference between samples stored in air at room temperature and in a vacuum drying oven (VDO) was small (Δ2.5°). This difference grew over time reaching a Δ24° lower CA for air. Over time, the CA steadily increased, more rapidly in a VDO than in air (Supplementary Materials, Figure S3). Consequently, all infiltrations were performed immediately after functionalization and subsequent bonding to minimize the opportunity for CA changes to occur. For the same reason, no devices were reused. 

Assembly: The bonding treatment of hydrophilized PDMS device parts led to a further decrease in CA to 37° and an increase in the polar *SFE* fraction to 44%, while total surface energy remained constant. The literature reports no significant influence of bonding on the CA [50]. Theoretically, plasma oxidation creates more hydrophilic functional groups on the surface of the PDMS, and the heating of the PDMS device parts during bonding should increase the probability of two reactive groups on the surfaces finding each other and bonding successfully [64]. Contrarily, by increasing the general mobility of polymer chains through heat, the process of hydrophobic recovery is also sped up [63]. For Demming, where the PDMS parts were not heated for bonding, these processes seemed to be balanced out, while for our reported treatment with heat during bonding, the increase in hydrophilicity seems to outweigh the hydrophobic recovery, at least in the short term [50].

Summary: Generally, a *PDMS* device can be manufactured (about 2 h), functionalized (about 3 h), bonded (30 min), and experimented on (30 min) within a day for a nearly unlimited number of prints from an existing master template. The modified *PDMS* before and after successful bonding can be considered hydrophilic and close in CA to that measured for water on calcite (55 ± 4°). Another indicator for the successful hydrophilization of the PDMS device parts is the fourfold increase in surface energy coupled with a 29% increase in its polar components. In general, the issue of hydrophobic recovery could be avoided by using devices directly after functionalization and assembly for infiltration experiments. 

#### 3.1.2. Poly(methyl methacrylate) Microfluidics

Microfluidic chips made from PMMA with straight channels and outlets on both ends were used as a commercial comparison system without further modification. They are available in various channel sizes and shapes, e.g., microscopy slide format chips. The CA of water on these chips is 75 ± 4° (Figure 6D), which is in good agreement with the literature (e.g., 77° [65]). Their main advantage is that they can be bought in desired quantities and are ready to use without further modification. Their rigid structure makes them very durable and easy to handle. When it comes to production time per device and reproducibility, commercially available PMMA chips present the most effective route, since their production is completely outsourced and standardized. Some limitations include available variations in channels, geometries, layouts, and materials—especially considering the incompatibility of PMMA with alcohols [24], rendering the chips damaged and unusable after a single infiltration with alcohols or alcoholic dispersions. 

#### 3.1.3. Glass–Resin Microcomb Test System

Hard lithography for glass–resin MCTS manufacture consists of similar steps as described for PDMS devices: photolithography, functionalization, and assembly. The difference is that while in soft photolithography the highly susceptible and variable process of thick-layer photolithography only has to be performed right once for a large number of imprints and resulting devices, it must be performed once per device when using the SU8 resin parts directly in the glass–resin MCTS. This results in greater variations in results between devices and significantly greater time investment. The expensive resin is so viscous that spin coating is not an option for transferring it to the substrate to yield reproducible film thicknesses, so doctor blading must be used. Worse still, the viscosity of the resin changes over time with evaporating solvents, and the parameters for reproducible results in device parts change continuously. Since the channel walls consist of glass slides on two sides (with a native CA of 41–55°) and resin (with a native CA of 93 ± 1°) on the other two, the surfaces must be somehow unified to present a consistent CA and wetting behavior. This necessitates an additional step of coating the device parts with gold (with a CA of 77 ± 2°) needed for the functionalization with thiol–gold-SAMs (with CAs as low as 30° possible [29]), increasing time investment. Additionally, the functionalization with these SAMs makes them vulnerable to oxidation and further process variation (because oxidized thiols lose their affinity for gold surfaces), introducing a further opportunity for variations. This method has the highest manufacturing time of all devices: 2–3 h for the lithographic process, 2–3 h for coating with gold, about 16 h for functionalization, and another 2 h for assembly and experimenting, plus an extra frame to hold everything together; needing to be completely repeated per device. 

For all of these reasons, the glass–resin MCTS was only investigated theoretically instead of experimentally, by modifying glass slides with gold coatings and thiol mixtures, as described in the literature. In this way, a water CA of 57 ± 4° (Figure 6E) was achieved for the coated and treated glass slides as a representation of the channel surfaces in the glass–resin MCTS, successfully imitating the CA of water on calcite (55 ± 4°) measured for this study (Figure 6F, in good agreement with previous literature [66,67]), as well as the CA previously achieved using this method in the literature [29].

### 3.2. Experimental Infiltration Study 

**Figure 7:**
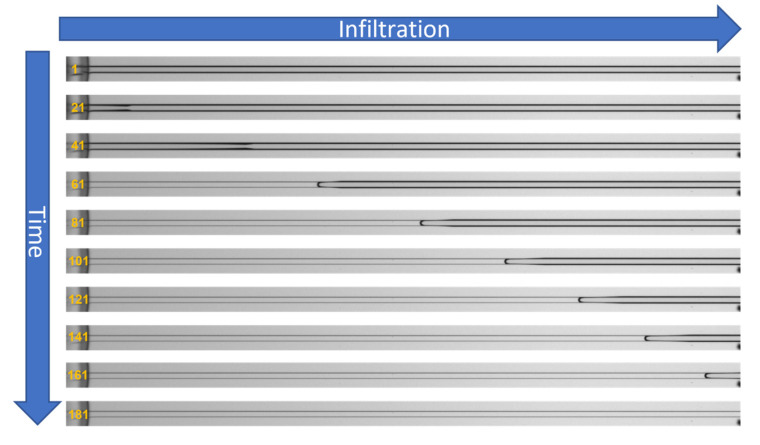
Light microscopic image time series showing meniscus progression *d* of a liquid into a channel from frame 1 to 181 in increments of 20 frames (Δ20 frames ≈ Δ0.1 s). Distance *d* traveled per frame decreases with time *t* and is linearly proportional to the square root of *t*.

**Figure 8:**
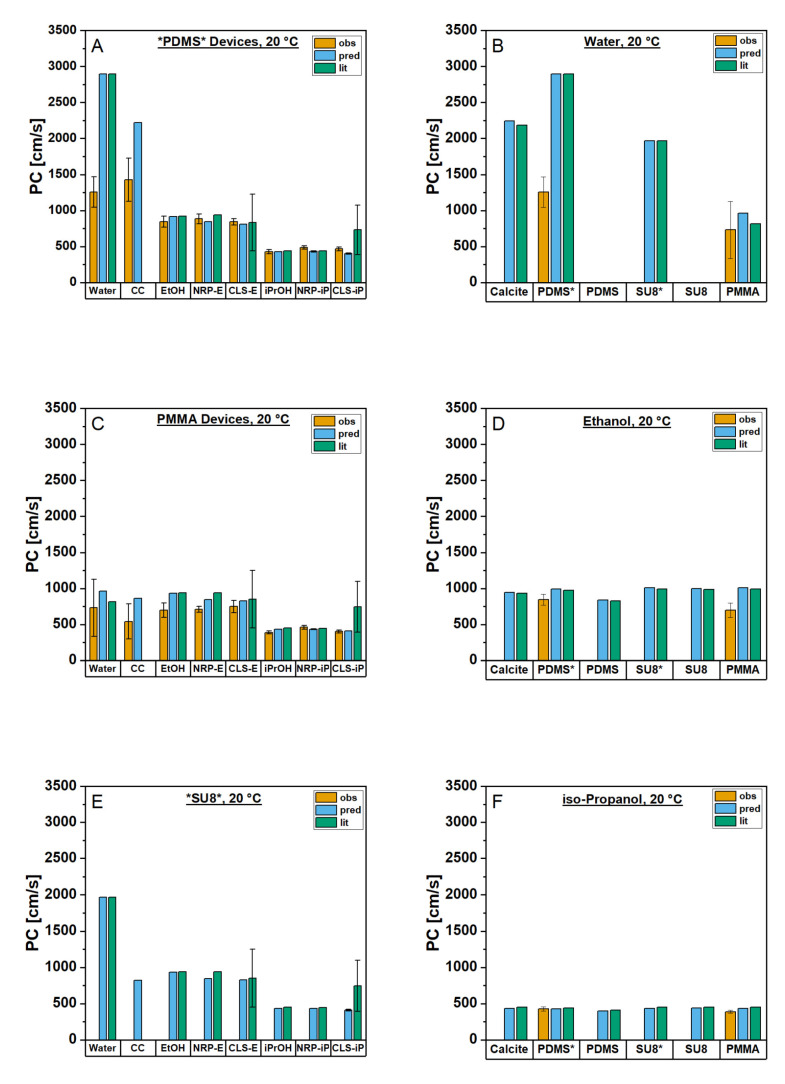
Observed (obs) and calculated (pred/lit) *PC*s for water, complex coacervate (CC), ethanol (-E, EtOH), isopropanol (-iP, iPrOH), Nanorestore Plus (NRP), and CaLoSiL (CLS) into modified *PDMS* (**A**) and native PMMA devices (**C**), as well as predicted and literature-based values for modified (coated) *SU8* (**E**). Additionally, *PC*s for solvents ((**B**): water; (**D**): EtOH; (**F**): iPrOH) into calcite, PMMA, modified (*), and native PDMS as well as modified (*) and native SU8 are displayed.

Here, the observed *PC* (*PC_obs_*) will be derived from experimental infiltration data of the liquids into the PMMA- and *PDMS*-based devices. To characterize their actual real-time infiltration behavior, they were placed on a light microscopy setup and observed. A series of images were recorded in time following the infiltration front of the solvents after they were applied to the device in an observation window of about 20 mm length from the entry point of the liquid. An exemplary infiltration image series are shown in Figure 7. The meniscus positions in the LM image series for every recorded time point were extracted [68] and can be plotted in different ways (Supplementary Materials, Figure S4). Examples are shown in the Supplementary Materials, Figures S5–S12. The most consistent results with the lowest standard deviations were obtained for distance *d* vs. square root of time *t* plots (Supplementary Materials, Tables S5 and S6). The results are also visualized in the left column in Figure 8 with the calculated results for *PC_pred_* and *PC_lit_* described in the following chapter.

The infiltration experiments in all devices were conducted as similarly as possible but did not work equally well with all samples and materials. PMMA chips offered the easiest setup, being used directly out of the box from the manufacturer. Trapped air bubbles or minor dust sometimes interfered with single measurement evaluations and monitoring. This could be compensated by repeated measurements. PDMS devices posed more difficulties with good timing needed to go straight from functionalization and assembly to infiltration with multiple devices. In very rare cases, leakages occurred, likely due to insufficient pressure during bonding. The opposite also occurred: due to excessive pressure during bonding, channel ends were sealed shut and the trapped air stopped infiltration, or water that had progressed through a different channel diffused back into the outlet of other channels of the same device, also ending meniscus progression prematurely. These channels were excluded or partially evaluated, if possible. For both PMMA and *PDMS*, water and complex coacervate (CC) infiltrations posed the greatest difficulty. This is likely due to water’s very high surface tension (73 mN/m) compared to alcohols (22 mN/m), which may be sufficient to resist the capillary suction and subsequently cause the lower than predicted observed *PC*s, and the greater variability in CAs with materials (Δ38°) compared to alcohols (Δ0–12°). The glass–resin MCTS from our previous works [29,35] could not be employed for observing infiltration to deduce the *PC,* as described in the Introduction and Results (Section 3.1.3). Summarily, the high time investment for manufacture, variability in quality, closed pore geometry, low durability of the components, and chemicals with varying properties over time, were the main reasons for it not being possible or even feasible to perform additional infiltration experiments in glass–resin MCTS (Supplementary Materials, Figure S13). The only advantage of the glass–resin MCTS that we are aware of is the proven capability for long-term monitoring of drying and crystallization inside semi-closed pore spaces with repeated infiltrations of dispersions [29,35], which is not possible for PMMA with alcohol-based dispersions (such as commercial NRP and CLS) and also not possible for *PDMS* due to its dynamic surface leading to hydrophobic recovery [63]. It is also a lot easier to disassemble than tightly sealed and rigid PMMA chips for analysis of the crystallized matter, and it is also more straightforward to extract crystals from glass/resin than from PDMS, which is rather sticky and soft.

The observed *PC* values for all devices exhibit similar behaviors (see Supplementary Materials, Tables S8 and S9). The dispersions in general show *PC_obs_* values similar to their respective solvents. For example, *PC_obs_* values for iPrOH in *PDMS* (427 cm/s) are in the same range as those of nanolime dispersions in this solvent (NRP: 486 cm/s; CLS: 466 cm/s). The same is true for EtOH (846 cm/s) and nanolime dispersions in EtOH (NRP: 885 cm/s; CLS: 843 cm/s) as well as the complex coacervate (CC) formulation (1428 cm/s) and water (1255 cm/s). Standard deviations are acceptable in the range of lower than 10% for all alcohols and alcoholic dispersions and up to about 20% for water and coacervates. *PC_obs_* values of solvents and dispersions in PMMA are consistently slightly lower than those in *PDMS* (149 cm/s lower for EtOH and 40 cm/s lower for iPrOH), with water and coacervates exhibiting only about half of their *PDMS*-*PC_obs_* value in PMMA (524 cm/s lower for water and 887 cm/s lower for coacervates) with considerably higher standard deviations (54% for water and 45% for coacervates). The other samples remain at more reasonable standard deviations between 5–14% in PMMA. Nevertheless, the similar range of *PCs* of dispersions and their solvents also applies here. 

An important factor influencing the *PC* value derived from experimental data is the capillary radius, which necessitates knowledge about the channel geometry of test systems or the pore structure of a natural stone sample to be treated. For square channels, which show a different behavior from circular channels [69], the area equivalent circle radius of the channel cross sections seems to be a good choice, as it represents a compromise between inner radius (*PC* overestimated due to underestimated radius/area) and outer radius (*PC* underestimated due to overestimated radius/area) of the rectangle, and the cross-sectional area is an important parameter for analyzing flow in any receptacle. The evaluation using the LW equation with the area equivalent radius gave the best results overall when compared to the calculated infiltration behavior, except for water and CC, whose *PC_obs_* in PMMA (~730 and ~540 cm/s) *PDMS* (~1255 and 1430 cm/s) seem to be systematically far too low compared to expected values (ca. 820–960 cm/s for PMMA and ca. 2099 cm/s for *PDMS*), therefore profiting from an underestimation of radius and area, i.e., an overestimation of *PC*.

In general, infiltrations into PMMA and *PDMS* devices with alcohols and alcoholic dispersions showed similar and repeatable curves if there were no disturbances present—e.g., bubbles or dust—while water and CC samples were prone to a lot of noise and stop and go, as well as unsuccessful infiltrations. Accordingly, some infiltration curves had to be reworked to remove outliers or signals from the disturbances detected by an ImageJ (FIJI) Makro [68] used for transforming image time series into time-dependent meniscus position data. Once disturbances were extracted, the evaluation of these infiltration experiments showed good agreement between plot types for each combination, which are the typical plots found in the literature for infiltration or capillarity data: infiltrated length *d* against time (LW Fit) or the square root of time (Linear Fit). *PC* values derived from plots against the square root of *t* agreed better with the literature and prediction-based values while the curves against *t* nicely illustrate the actual infiltration profile preset in the microscopy images, which is why they were used for comparison of simulated curves in the next chapter.

### 3.3. Theoretical Infiltration Studies

As a comparison for the *PC*_obs_ values, calculations of *PC*s according to Equation (2) were executed. These calculations were run with two different input sources: one based on the literature values (*PC_lit_*) and the other predicted from experimentally determined physicochemical properties of the solvents and dispersions (*PC_pred_*). The relevant solvent and dispersion parameters for the calculations have been published in a data repository [70] (summarized in Supplementary Materials, Table S7). Calculated results for *PC_lit_* and *PC*_pred_ are illustrated in Figure 8 along with the results for *PC*_obs_ (exact values: Supplementary Materials, Tables S8 and S9).

These results also show similar trends for all devices. In general, solvents and their dispersions displayed similar *PC_lit_* values except for CLS-iP, which exhibited a comparatively high *PC_lit_* value with a significant error margin (488–992 cm/s) due to the broad range of literature-given *η* values of 1–2 mPas. The lower end of these literature-based values, though, can be considered in the same range as the *PC_lit_* of NRP–iP (442–443 cm/s) and pure iP (440–511 cm/s). The range of *η* values used for the *PC_lit_* of CLS-E (555–1134 cm/s) was a comparably better fit for the *PC_lit_* values of NRP-E (940 cm/s) and pure EtOH (921–992 cm/s). The same trends could be observed for the *PC_pred_* results, as the agreement between the two was generally good. In *PDMS*, the differences range from 1 cm/s for water to 98 cm/s for NRP-E. PMMA exhibits slightly greater deviations between 3 cm/s for EtOH and 146 cm/s for water. For the glass–resin microcomb test system, the values deviate by 1–98 cm/s. The only significant differences between solvent and dispersion were observed for the coacervates: they were predicted to be significantly (SU8: Δ1150 cm/s; *PDMS*: Δ677 cm/s) or slightly (PMMA: Δ98 cm/s) lower in *PC* than water, its solvent. When comparing *PC_lit_* and *PC_pred_* values across devices, values for alcohols and alcoholic dispersions are rather similar even in absolute terms, while the largest and only significant difference between devices was observed for water and CC formulations. Here, the *PC_pred_* and *PC_lit_* values are about three times higher in *PDMS* than PMMA, while SU8 shows values similar to PMMA for CC and a *PC* value between the other two device types for water. 

Simulated infiltration curves could be generated and compared to reference materials by reentering the calculated and observed *PC*s into the LW equation [53,54] (Equation (1), see Supplementary Materials, Figures S14–S16). All trends observed for the *PC* values can also be observed in their simulated curves since they are the product of multiplication with a constant factor (radius *r*). 

The quality of the *PC* calculations and subsequent simulations based on the literature or experimentally determined material properties of our samples depend strongly on the quality of the input data. In the case of available literature data on water, EtOH, and iPrOH, we can assume this to be sufficiently high. For example, static CAs of unaltered PMMA, PDMS, and some SU8 types are given in the literature as 100–110° water CAs for PDMS, 77° for PMMA, and 72° for SU8-50. [65] Measurements of the untreated substrates resulted in about 113° for PDMS, 77° for PMMA, and 93° for SU8-3050. Apart from the different SU8 types, all of these values are in good accordance with each other. Since the experimental data collected during this study are in good agreement with the literature and exhibit relatively low standard deviations where applicable, we can assume that their quality is also sufficient. Therefore, the calculated *PC* values and simulated curves should also be of good quality. This is further proven by the changes in all *PC* values and curve shapes corresponding to the influence certain properties should have on them according to the LW equation. For example, water has a much higher *PC* and accordingly a much steeper infiltration curve than the alcohols due to its higher surface tension (73 vs. ~20 mN/m for alcohols) and CAs with the materials of the devices, leading to higher values in the numerator of the fraction defining the *PC*. While the viscosities of EtOH and water are quite similar, that of iPrOH is much higher, with 2.4 mPas compared to 1.0 mPas, decreasing its *PC* value compared to water and EtOH further. CCs have surface tensions and CAs very similar to water, but a slightly higher viscosity due to forming dense liquid phases in the aqueous phase. This explains their similar but slightly lower *PC* values and infiltration curves. 

Interestingly, the differences between devices for the respective liquids are almost negligible for alcohols and their dispersions but very pronounced for water and CCs. This is most likely due to the CAs of water differing more strongly between *PDMS* and PMMA (Δ38°), while the CAs of EtOH and iPrOH (and their dispersions) are not as strongly influenced by the material of the device (Δ0–12°). SU8 exhibits alcohol CAs identical to PMMA, while its CA with CC matches PMMA, but the water CA is situated between PMMA and *PDMS* devices. Therefore, theoretically, the glass–resin MCTS should be just as capable as PMMA devices for the observation of alcoholic infiltrations when considering its material properties and may be better suited for water infiltrations with a rather midrange CA.

Overall, the Lucas–Washburn equation was demonstrated to successfully predict qualitative trends in the infiltration behavior of solvents and nanolimes as well as CCs for test systems made of different materials (PMMA and *PDMS*), even though the experimental setup deviates from the prerequisites defined for it (horizontal round capillaries with unlimited liquid reservoir) [53]. For alcohols, the absolute predicted and literature-based *PC* values come very close to those derived from observed infiltration behavior. The observed *PC*s being consistently lower than predicted and literature-based *PC*s is most likely due to the presence of liquid fingers in the corners of the square capillary tube preceding the bulk meniscus, which can slow down infiltration by about 3% compared to Lucas–Washburn behavior according to Yu et al. [69]. Additional possible factors are roughness and friction. Additionally, the *PC* values of all liquids in all materials are high enough to be considered very good infiltrants, as defined by Paris et al. (*PC* > 100 cm/s) [71]. It is important to note that we have investigated horizontal infiltration using thin micromodels in this study without considering the influence of gravity. If artifacts are not infiltrated horizontally or thick enough that gravity will play a role, predictions must very likely be adjusted before they can be applied, because fluids in porous media under the influence of gravity behave differently [72]. Glass–resin MCTS (modified SU8) are theoretically better suited for the infiltration of alcohols than PMMA and potentially also for water when only considering their material properties and calculated *PC* values. Their drawbacks, including geometry, effectively prevent this. A solution could be the stereolithographic 3D printing of the structured part of the micromodel [73], although often resolutions of available 3D printers are not high enough for channels of 100 µm side lengths and smaller. 

### 3.4. Penetration Coefficients of Calcite

Comparing calculated *PC*s and simulated curves of our reference material, calcite, to the device materials (see Figure 8 right column), it is noteworthy that the hydrophilization of the native PDMS and SU8 materials brings the resulting *PC*s and simulations for water-based liquid infiltration closer to the results obtained for the mineral, as desired. With the modified PDMS (*PDMS*), we have overshot the goal due to our bonding step further hydrophilizing the material below the desired 50°, resulting in greater *PC* values and steeper curves. The observed curve and *PC* lie between PMMA and *SU8* values, indicative of other factors influencing the infiltration or an increase in CAs above 50° after bonding. For EtOH (center row) and iPrOH (bottom row), *PC* values and simulations were very similar, sometimes even equal. This reinforces the observation that alcoholic infiltrations (and the underlying contact angles) are nearly material independent, certainly more than water infiltrations.

Calculations of converted *PC*s (*cPC*) according to Equations (3) and (4) were only executed for solvents using *PC_pred_*, and not for their dispersions or *PC_lit_* due to their similarity. Ideally, the converted c*PC* should be identical to the *PC_obs_* and *PC_pred_* (or *PC_lit_)* reported earlier. To test the conversion capability and then employ it, several conversions were performed:

- Between *PDMS* and PMMA for identical liquids with Equation (4) (Supplementary Materials, Tables S10–S2);
- Between different liquids for identical materials with Equation (3) (Supplementary Materials Tables S13–S16);
- From *PDMS* and PMMA to calcite for identical liquids with Equation (4) (Table 1 and Table 2);
- Between arbitrary liquid/device combinations, into water/calcite c*PCs* (Table 1 and Table 2).

For example, the *cPC* of water in PMMA can be determined using the *PC* of ethanol in *PDMS*:

(5) $$cPC_{Water/PMMA}\;\left(\mathrm{EtOH}/*\mathrm{PDMS}*\right)=PC_{EtOH/*PDMS*}*\frac{\sigma_{W}*\cos\theta_{W/PMMA}*\eta_{E}}{\sigma_{E}*\cos\theta_{E/*PDMS*}*\;\eta_{W}}=963\;\mathrm{cm}/s$$

which is identical to the predicted *PC* of water in PMMA. Therefore, we can assume that this is generally possible and convert all combinations of observed and predicted *PC* values in *PDMS* and PMMA into a *PC* for calcite and water, our desired reference target system (Table 1 and Table 2).

For conversions from the same liquids between PMMA and *PDMS* using *PC_obs_*, alcohols yielded identical results while water and coacervates did not convert well, indicating stronger interference on practical infiltrations for aqueous than alcoholic systems. For *PC_pred_* values and experimentally determined *CAs*, the conversions between PMMA and *PDMS* for all solvents and CC was excellent. Mixing *PC_obs_* with experimentally determined CAs yielded the worst results and will, therefore, not be considered further. This can be explained by the difference in experimentally measured contact angle and that calculated backward from the observed *PC*s: the most significant difference occurred with calculated CAs of 70–77° for water and 64–73° for CCs in *PDMS* (depending on whether the liquid parameters used for calculation were measured or taken from the literature), while measured contact angles were only around 37° for water and 42° for CC. This explains why *PC_obs_* is only about half of *PC_pred_* and *PC_lit_* for *PDMS* with water and the coacervates. Obviously, some practical deviations must be occurring during infiltrations, e.g., inhomogeneous wetting, cohesion, decrease in channel cross section, or friction. For PMMA, on the other hand, the calculated and measured contact angles are basically identical for water and coacervate infiltrations, explaining the good agreement of *PC_obs_* with *PC_pred_* and *PC_lit_*. Here, the influence of the wetting properties on the deviations is clearly less pronounced than in *PDMS*. For alcohols, the calculated CAs do not differ significantly from the measured CAs, considering the increasing inaccuracy and difficulty of measuring contact angles below 30°. Further, alcohols have a damaging effect on the PMMA, presumably causing micro ruptures and consequently influencing spreading behavior on the surface. For CLS dispersions, deviations are most likely also due to the very imprecise literature-based value of its viscosity (1–2 mPas).

When converting different liquids into each other for the same device material, the trends observed were similar to the observations made for the same liquids and differing materials, except for the observed values of *PC* in PMMA giving good conversion values for water and CC. This is most likely due to the better accordance of *PC_obs_* values for water in PMMA with the calculated counterparts than those of water in *PDMS*, which shows the worst agreement with observed values being only 43% (water) or 64% of calculated values. CC in PMMA fares even worse at 62%, but water in PMMA reaches agreements of 76% with *PC_pred_* and 90% with *PC_lit_*. Best agreements are achieved for alcohols in *PDMS*, ranging from 92–100%, while alcohols in PMMA make it to about 75% (EtOH) and about 88% (iPrOH). The almost identical decrease in *PC_obs_* vs. calculated *PC*s for CC could be coincidental or could point to a practical reduction factor for water or coacervates of about 40–45%. The slightly worse agreements between *cPC* for PMMA and alcohols with the original *PC*s could be attributed to the alcohol reacting with and damaging the PMMA, resulting in faulty or imprecise contact angle measurements used for the conversion of the *PC_pred_* values.

The conversion results for predictions of calcite/liquid *PC*s yield perfect agreement with each other when using the PMMA *PC_obs_* values as the basis. For the *PC_pred_* values based on PMMA, slight deviations occur for iPrOH that can be considered negligible (9%). What is even more remarkable is that all values agree well for both types of *PC* as the basis. This is also true across device materials used as the basis for conversions: both the PMMA and the *PDMS* basis yields a conversion result of 2244 cm/s for water, 1964 cm/s for CC, 876 cm/s for ethanol, and 436 cm/s for isopropanol (highlighted in green in the results tables). Only conversions based on *PC*s of water or CC in *PDMS* deviate significantly, resulting in values about 50–60% higher than those predicted by all other variations. Apart from that, only iPrOH conversions for identical liquids from *PDMS* to Calcite show even a slight deviation (439 cm/s, up 3 cm/s from 436 cm/s). These deviations are highlighted in the results tables in yellow.

In summary, the calculation and conversion of *PC*s can be considered successful and brings with it many potential benefits:

- It is a most promising method for the predictions of well-agreeing *PC*s for calcite/liquid systems using measured or calculated *PC*s of any other liquid/material combination as the basis.
- For reliable conversion, the basis used must be solid and of good quality. In this case, alcoholic measurements in *PDMS* were the best measurements followed by alcohols and water in PMMA.
- In this way, the Lucas–Washburn equation can be used to predict identical or near-identical calcite *PC*s for a given liquid from various other material/liquid combinations.
- It is strongly indicated that this is generally a suitable prediction method for infiltration behavior using *PCs*, maybe needing a practical adjustment factor for water and CC of about 50–60% (according to prediction differences and differences in measured and recalculated CAs from observed *PC*s).
- This way, we can now make predictions for nontransparent systems using experimental data obtained from transparent systems as the basis to aid the targeted design of (for example) stone treatments.

## 4. Conclusions

When comparing all three investigated device types, the following can be said:

- The commercially available acrylic (PMMA) chips seem the least time consuming and most reproducible in terms of manufacturing, yielding good infiltration results compared to literature calculations, and experimental predictions, with some restrictions such as limited commercially available designs, but most notably without the necessity for a modification step.
- This chip is closely followed by the silicon polymer (PDMS) device: its advantage lies in the possibility of custom designs with low-cost mass production capabilities and only one photolithographic master required for many PDMS imprints [55]. Drawbacks are the strongly hydrophobic nature of PDMS [50], which necessitates a functionalization step for the infiltration of most solutions, as well as its swelling behavior with many organic solvents [74]. Furthermore, the fabrication of master templates requires specialized equipment.
- The glass–resin MCTS in its current published form is not suitable for *PC* determination but could be, based on calculations of predicted and literature-based *PC*s, if open pore designs can be realized and manufacturing improved.

For calculations of Penetration Coefficients (*PC*s) with the Lucas–Washburn equation, the following results were obtained:

- It is possible to predict trends in the observed infiltration behavior at least qualitatively. This is in agreement with literature reports on the infiltration of caries lesions with monomer mixtures [52,71] and adhesives [75].
- The exact prediction of absolute values proves challenging, especially for water-based systems.
- The similarity between all *PC* values for the alcoholic systems as well as their good quantitative agreement is noteworthy.
- As long as a liquid achieves the same contact angle with the material as the reference (here: calcite), a semiquantitative infiltration prediction can be made for alcoholic systems, while more research and fine-tuning are needed for water-based systems.
- A very promising result is the excellent agreement of converted *PC* values for calcite/liquid systems based on the Lucas–Washburn equations using either observed or predicted *PC* values in modified PDMS (*) or native PMMA for all liquids except water and CC in *PDMS*, where an experimental adjustment factor might be needed, estimated to be around 50%.

Using experimental and calculated *PC* data from transparent systems, we can now make predictions for the *PC*s of any desired (also nontransparent) systems such as a stone for restoration treatments. Two remaining open questions must be addressed in further research:

- Are the observations made for these micromodel and liquid combinations transferable to real stone and relevant for restoration? For example, marble and sandstone, as imitated by Gruber et al. [29], calcite, as imitated here, or other calcium carbonate-containing minerals. While the *PC* as such is independent of pore radius *r*, it remains to be seen if it is suitable for describing more complex porous systems such as natural stone.

If this were the case, destructive sampling for case studies could be minimized, necessary testing reduced, and solutions rapidly tailor-made for each object faced in the field by targeted design based on these infiltration behavior predictions from experimental micromodel data. Since Paris et al. [71] were able to demonstrate the applicability of the Washburn equation to predict resin infiltration into enamel lesions and saw a good correlation between penetration coefficients and penetration depth, this is a very promising endeavor.

- Are complex coacervates able to effectively consolidate and remineralize deteriorated stone samples, ideally without introducing further problems?

A nonhazardous, stone-compatible remineralizing agent that is easy to manufacture on-site and not limited by pore sizes would be a tremendous innovation for saving large parts of humankind’s cultural heritage. To avoid introducing harmful substances into treated objects, a salt-free formulation must be developed before it can be used in the field for real case studies on-site.

When further pursued successfully, this micromodel-based prediction system has the potential to save many hours of trials (and, therefore, also money), damage to artifacts by destructive sampling, and can help avoid unsuitable treatments in the future.

## Figures and Tables

**Table 1:** *cPC*s for calcite and water, CC, EtOH, iPrOH—converted from *PDMS* and PMMA *PC*_obs_ values, and derived CAs and experimental properties. Good agreement: green. Deviation: yellow.

Based on: *PC*_obs_	*PDMS* with				PMMA with			
	Water	CC	EtOH	iPrOH	Water	CC	EtOH	iPrOH
*cPC_Calcite_* with:								
Water	3328	3624	2244	2244	2244	2244	2244	2244
CC	2912	3171	1964	1964	1964	1964	1964	1964
EtOH	1300	1415	876	876	876	876	876	876
iPrOH	646	703	436	436	436	436	436	436

**Table 2:** *cPC*s for calcite and water, CC, EtOH, iPrOH—converted from *PDMS* and PMMA *PC*_pred_ values and experimental properties. Good agreement: green. Deviation: yellow.

Based on: *PC*_pred_	*PDMS* with				PMMA with			
	Water	CC	EtOH	iPrOH	Water	CC	EtOH	iPrOH
*cPC_Calcite_* with:								
Water	2244	2244	2244	2244	2244	2244	2244	2051
CC	1964	1964	1964	1964	1964	1964	1964	1795
EtOH	876	876	876	876	876	876	876	801
iPrOH	436	436	436	439	436	436	436	398

## Data Availability

Raw data obtained during the infiltration experiments with a light microscope as well as meniscus positions and times extracted using the ImageJ Macro [68] are available on KonDATA [76,77] as part of a parent data package for this paper [78]. Rheometry, viscosity, density, and surface tension data are also available there [70]. Literature data of liquid properties used for calculations were taken from the following sources: [79,80,81,82,83,84,85,86,87].

## Supplementary Materials

The following supporting information can be downloaded at: https://www.mdpi.com/article/10.3390/ma16062506/s1, information about used instruments, details on evaluation of infiltration curve data and penetration coefficient determination from observed experiments, details on calculations and simulations of penetration coefficients and infiltrations, further calculations for calcite penetration coefficient predictions, error calculation methods, Videos S1–S3 (infiltration observation via LM for ethanol, iso-propanol, and water into modified PDMS devices). Table S1: Height, basal width and plateau width for 17 protruding structures of the MT on Si-Wafer; Table S2: Mean height, basal width, plateau width and channel cross section area for the mastertemplate; Table S3: Height, basal width, plateau width of 17 channels of PDMS imprint of the MT from LM; Table S4: Mean height, basal width, plateau width and channel cross section area for the PDMS imprint; Table S5: Observed penetration coefficients in PDMS devices; Table S6: Observed penetration coefficients in PMMA; Table S7: Summary of solvent properties and contact angles used for this study; Table S8:Penetration coefficient results for modified PDMS, native PMMA, and modified SU8; Table S9: Penetration coefficient results for Calcite, native PDMS, and native SU8; Tables S10–S12: Converted penetration coefficients for conversions between modified PDMS and native PMMA for identical liquids; Tables S13–S16: Converted penetration coefficients within the same material with different liquids; Figure S1: Mask structure used for master template photolithography and profile of the resulting master template; Figure S2: Schematic imprint and master template with cross sections and structure parameters visualized, additionally a light microscopic channel image in PDMS; Figure S3: Bar chart of contact angles for water on functionalized PDMS in air or vacuum drying oven from 0 to 6 days; Figure S4: 3 different infiltration data plot possibilities with their approximation methods; Figures S5–S12: Various plotted experimental infiltration curves of different liquids into different materials; Figure S13: Light microscopic images of SU8 resin-based test systems highlighting issues; Figures S14–S16: Simulated infiltration curves for various material and liquid combinations.
