# Supplementary material for: Penetration Coefficients of Commercial Nanolimes and a Liquid Mineral Precursor for Pore-Imitating Test Systems—Predictability of Infiltration Behavior
Source: Materials (Basel). 2023 Mar 21;16(6):2506. doi: 10.3390/ma16062506 (PMC10058312; doi:10.3390/ma16062506)
Supplement: Supplementary file 1 [file materials-16-02506-s001.zip › materials-2139762-supplementary.pdf]

materials

MDPI

Article

Penetration Coefficients of Commercial Nanolimes and a Liquid Mineral Precursor for Pore-Imitating Test Systems—Predictability of Infiltration Behavior

Andra-Lisa M. Hoyt, Marc Staiger, Marcel Schweinbeck and Helmut Cölfen *

Department of Chemistry, Physical Chemistry, University of Konstanz, Universitätsstraße 10, 78457 Konstanz, Germany; Andra-Lisa.Hoyt@uni-konstanz.de (A.-L.M.H.)
* Correspondence: Helmut.Coelfen@uni-konstanz.de; Tel.: +49-7531-88-4063

Abstract: Nanolimes have been commercially available for over a decade as a remineralization agent for natural stone to combat deterioration. While they have been applied successfully and studied extensively, their penetration abilities in different materials have not yet been readily quantifiable in situ and in real-time. Using two transparent pore-imitating test systems (acrylic glass (PMMA), polydimethylsiloxane (PDMS)) and light microscopy, the penetration coefficients (*PCs*) of two nanolimes (CaLoSiL (CLS) and Nanorestore Plus (NRP)), as well as their solvents, were determined experimentally in square channels of about 100 µm diameter. Their *PCs* and those for a previously published glass-resin-based test system were also predicted based on measurable material parameters or literature values using the Lucas-Washburn equation. Additionally, a liquid mineral precursor (LMP) of calcium carbonate based on complex coacervation (CC) was investigated as an alternative to the solid particle dispersions of nanolime. In general, the dispersions behaved like their pure solvents. Overall, trends could be reasonably well predicted both with literature and experimentally determined properties using the Lucas-Washburn equation. In absolute terms, the prediction of observed infiltration behavior was satisfactory for alcohols and nanolimes but deviated substantially for water and the aqueous LMP. The commercially available PMMA chips and newly designed PDMS devices were mostly superior to the previously published glass-resin-based test system, except for the long-time monitoring of material deposition. Lastly, the transfer of results from these investigated systems to a different, non-transparent mineral, Calcite, yielded similar *PC* values independently of the original data used as basis for the conversion (all *PC* types and all material/liquid combinations except aqueous solutions in PDMS devices). This knowledge can be used to improve targeted design of tailor-made remineralization treatments for different application cases by guiding solvent choice, and to reduce destructive sample taking by providing a micromodel for pre-testing, if transferability to real stone samples proves demonstrable in the future.

Keywords: complex coacervation; nanolime; calcite; liquid mineral precursor; porous material; infiltration; remineralization; Lucas-Washburn equation; capillarity

Citation: Hoyt, A.-L.M.; Staiger, M.; Schweinbeck, M.; Cölfen, H. Penetration Coefficients of Commercial Nanolimes and a Liquid Mineral Precursor for Pore-Imitating Test Systems—Predictability of Infiltration Behavior. *Materials* **2023**, *16*, 2506. https://doi.org/10.3390/ma16062506

Academic Editors: Jorge Otero, Luis Monasterio-Guillot and Giovanni Borsoi

Received: 19 December 2022
Revised: 7 March 2023
Accepted: 13 March 2023
Published: 21 March 2023

Copyright: © 2023 by the authors. Licensee MDPI, Basel, Switzerland. This article is an open access article distributed under the terms and conditions of the Creative Commons Attribution (CC BY) license (https://creativecommons.org/licenses/by/4.0/).

Supplementary Information Document with more information on the following topics:

I. Instruments
II. Soft Photolithographic Route
   a. Characterization of the Master Template and PDMS Imprint
   b. PDMS Functionalization Stability
III. Experimental Infiltration Studies
   a. Method for the Evaluation of Infiltration Curves
   b. Resulting Observed Penetration Coefficients
   c. Experimental Infiltration Curves
   d. Glass–Resin Micromodel Issues
IV. Theoretical Infiltration Studies

a. Relevant Liquid and Material Properties
b. Simulations of Infiltration Curves
V. Results for Penetration Coefficients
VI. Predictions for Calcite
VII. Error Calculations

I. Instruments Used

Coating of silicon wafers with SU8 was done using an *OPTIcoat SB20+* spin-coater from SSE (ATM Group, Degotec GmbH, Singen, Germany). Photo resins were exposed to 365 nm (i-line) UV-light with a mercury gas lamp and an *MJB3 Mask Aligner* from Süss MicroTec AG (Garching, Germany). An *ATC Orion 5-T* thermal evaporation system from AJA International Inc (North Scituate, MA, USA) was used for film deposition of Cr and Au. Two-inch Cr plated tungsten rods and 1-3 mm Au granules were purchased from Testbourne Ltd. (Hampshire, UK). The mask for photolithography was manufactured by Compugraphics Jena GmbH (Jena, Germany) for chromium-glass and Selba S.A. (Geneva, Switzerland) for flexible plastic masks (see SI for designs). Light microscopy images were recorded on an *AxioZoom.V16* from Zeiss (Oberkochen, Germany). Their *ZEN blue* software and *FIJI (ImageJ)*[1] was used for image processing. Density measurements were performed using a *Density Meter DMA 5000M* from Anton Paar GmbH(Graz, Austria). Viscosities were determined using a *Discovery HR-2 hybrid rheometer* from TA instruments (Hüllhorst, Germany) or a *Lovis 2000 Viscosity Module* from Anton Paar GmbH (Graz, Austria). Contact angle (CA) and surface tension measurements were executed with a *DropShapeAnalyzer DSA25* from Krüss GmbH (Hamburg, Germany) using original accessories (*NE83, NE45 and NE44 needles; 1 mL syringes*). Their data was evaluated using the included software *Advance*. pH values were measured by a Metrohm Schweiz AG *691 pH meter* (Zofingen, Switzerland). PDMS activation for functionalization was performed with the *plasma oxidizer SI 220* from SENTECH Instruments GmbH (Berlin, Germany), while PDMS activation for bonding was done using a *Femto model 1A type A* from Diener electronic GmbH + Co. KG (Ebhausen, Germany). Surface profiles were recorded with a *DektakXT Advanced System* from Bruker Corporation (Billerica, MA, USA) with a *Stylus 2 µm, B-Type, Red/White*.

II. Soft Photolithographic Route

II.I. Characterization of Master Template and PDMS Imprint

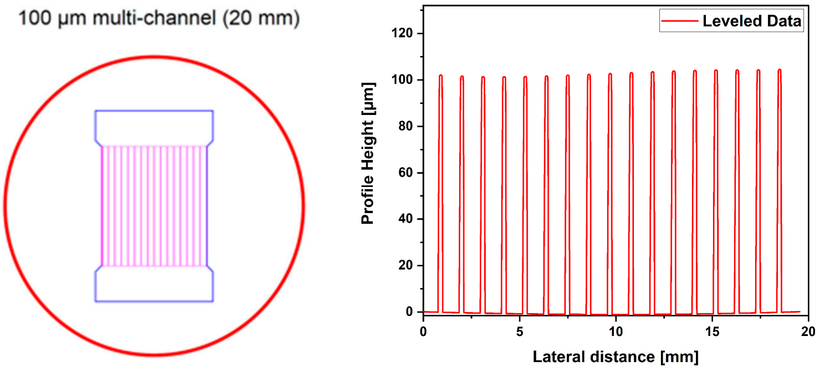

Figure S1. Left - Scheme of mask structure used for the MT on Si-Wafer (AutoCAD Student Version). Diameter of the red ring ~ 3 cm. Channel length = 2 cm. Right - Profile of the MT (contact profilometer with a 2 µm stylus).

Table S1. Values for height, basal width and plateau width for 17 protruding structures of the MT on Si-Wafer.

Structure No.	Height H [µm]	Basal Width wB [µm]	Plateau Width wP [µm]
**1**	102.09	252.20	117.74

**2**	101.62	254.81	119.26
**3**	101.34	256.33	116.87
**4**	101.33	254.59	117.52
**5**	101.43	250.90	115.13
**6**	101.65	248.29	115.35
**7**	102.00	248.51	114.91
**8**	102.37	245.90	113.61
**9**	102.72	251.33	114.04
**10**	103.11	253.94	119.47
**11**	103.47	251.33	114.91
**12**	103.82	256.11	118.39
**13**	104.05	253.50	117.30
**14**	104.22	250.68	113.83
**15**	104.27	252.63	117.30
**16**	104.33	248.94	112.09
**17**	104.55	245.90	112.52
**Mean:**	**102.85**	**251.52**	**115.90**
**σ:**	**1.16**	**3.13**	**2.21**

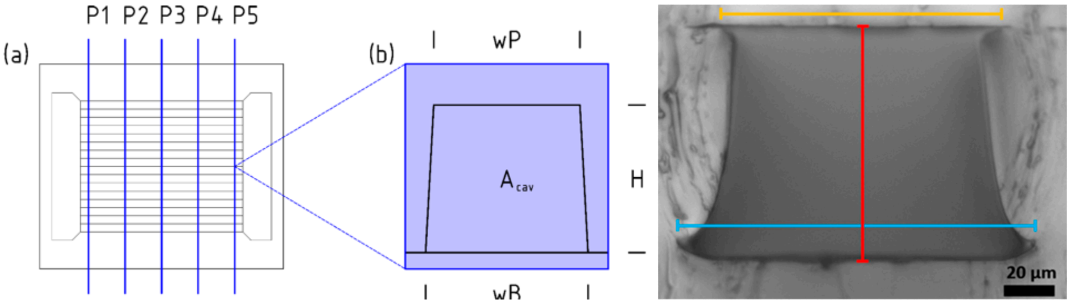

Figure S2. Left - Schematic imprint/MT with cross sections P1-5. (a) schematic from top with cross section planes P1-5 in blue. (b) schematic channel cross section with height H, basal width wB, plateau width wP, area A_{cav} (LibreCAD). Right - Light microscopy image of channel cross section in a PDMS imprint with H, wB and wP .

Table S2. Mean values with standard deviations in brackets for height, basal width, plateau width and channel cross section area of profiles P1-5 and average measured perpendicular to the channel structures of the MT with a mechanical contact profilometer.

Profile No.	Height H [µm]	Basal Width wB [µm]	Plateau Width wP [µm]	Area A_{cav} [µm^2]
**1**	88.6 (0.8)	223.2 (3.3)	113.1 (3.3)	14 899 (266)
**2**	92.8 (2.0)	227.1 (4.8)	112.0 (2.2)	15 737 (567)
**3**	90.7 (3.1)	226.8 (4.9)	113.4 (3.8)	15 433 (670)
**4**	89.2 (3.6)	224.8 (3.5)	112.7 (3.5)	15 055 (704)
**5**	86.4 (3.6)	225.7 (6.1)	115.6 (3.6)	14 751 (762)
**Mean:**	**89.5**	**225.5**	**113.3**	**15 178**
**σ:**	**3.6**	**4.9**	**3.5**	**720**

Table S3. Values for height, basal width, plateau width of 17 channels of PDMS imprint of the MT from LM.

Structure No.	Height H [µm]	Basal Width wB [µm]	Plateau Width wP [µm]	Area A_{cav} [µm^2]
**1**	92.889	108.051	136.087	10036.75

**2**	92.738	109.571	140.097	10161.40
**3**	92.656	110.157	138.606	10206.71
**4**	92.774	108.432	140.090	10059.67
**5**	92.271	113.433	141.741	10466.58
**6**	90.888	110.909	142.794	10080.30
**7**	92.500	113.413	138.550	10491.16
**8**	90.567	110.491	139.320	10006.84
**9**	91.528	112.898	139.315	10333.33
**10**	91.677	118.000	142.994	10817.89
**11**	91.657	113.947	140.466	10444.04
**12**	91.849	110.297	141.042	10130.67
**13**	91.515	116.504	144.383	10661.86
**14**	94.156	111.481	141.050	10496.61
**15**	92.235	115.753	143.996	10676.48
**16**	91.708	116.117	142.058	10648.86
**17**	94.713	110.352	137.481	10451.77
**Mean:**	**92.25**	**112.34**	**140.59**	**10362.99**
**σ:**	**1.05**	**2.97**	**2.27**	**257.49**

Table S4. Mean values with standard deviations in brackets for height, basal width, plateau width and channel cross section area of each of the profiles P1-5 and their average measured perpendicular to the channel structures of the PDMS imprint with light microscopy images. Mean values and standard deviations of single profiles were determined from 17 channels each.

**Profile Cut No.**	**Height H [μm]**	**Basal Width wB [μm]**	**Plateau Width wP [μm]**	**Area A_{cav} [μm^2]**
**1**	84.1 (1.4)	117.2 (2.1)	116.9 (1.5)	9 843 (240)
**2**	90.1 (3.0)	116.8 (1.9)	116.5 (1.9)	10 507 (369)
**3**	88.5 (4.1)	116.2 (1.4)	115.7 (1.7)	10 261 (475)
**4**	84.7 (2.9)	117.9 (4.3)	115.9 (6.1)	9 904 (510)
**5**	89.2 (3.9)	115.8 (2.4)	115.6 (1.8)	10 317 (387)
**Mean:**	**87.2**	**116.9**	**116.2**	**10 155**
**σ:**	**4.0**	**2.8**	**3.3**	**481**

II.II. PDMS Functionalization Stability

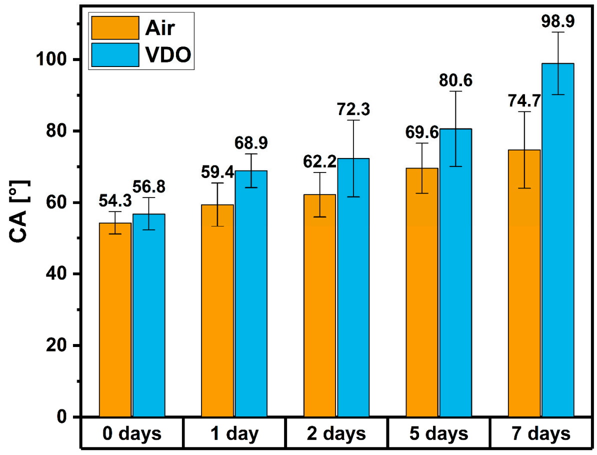

Figure S3. Bar chart of long-term stability of PDMS functionalization, measured by CA with water from day of treatment (0 days) up to seven days later depending on storage in ambient air or a vacuum drying oven (VDO).

III. Experimental Infiltration Studies

III.I. Method for Evaluation of Infiltration Curves

From these plots, *PC*s can be derived: plots of d vs t were fitted with the Lucas-Washburn equation, yielding an approximation for $PC * r$. Dividing this by the pore radius r of the investigated system yields the observed penetration coefficient PC_{obs}. Plots of d^2 vs t and d vs $\sqrt{t}$ were linearly approximated, where the slope of the former divided by r yields PC_{obs} and the latter slope divided by r needs to be squared to give PC_{obs}. For PMMA devices we can assume a rectangular channel with 100 µm*100 µm dimensions and an according inner, outer, and area equivalent circle radius r (50, 70.71, and 56.42 µm, respectively). The mean value with standard deviation for *PCs* calculated using the area equivalent radius is given as PC_{obs} result. PDMS devices were handled the same way because their equivalent circle radius is only marginally different (57.43 µm).

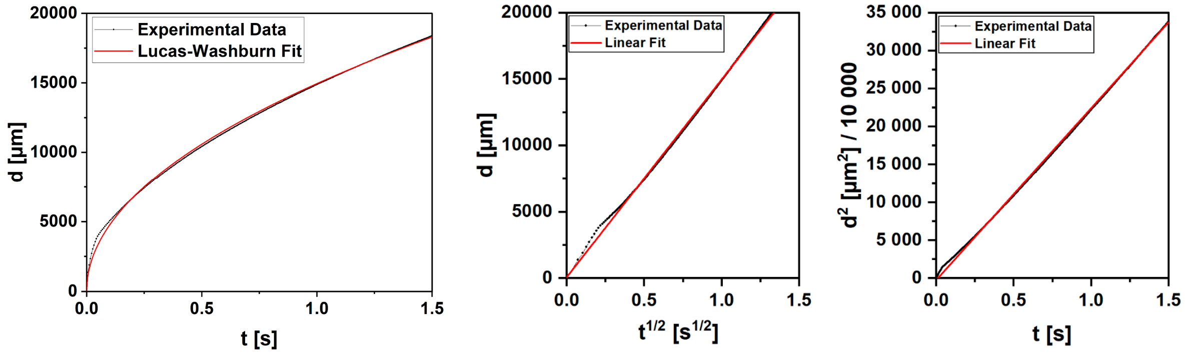

Figure S4. Different plot possibilities for infiltration data obtained from experimental observations of the devices under the light microscope with their respective fits. Left: meniscus position d vs time t plot fitted with the Lucas Washburn equation. Middle: d vs. the square root of t, with a linear approximation. Right: The d^2 vs. t plot, linearly approximated.

III.II. Resulting observed Penetration Coefficients (PC)

For each infiltrated channel, the data was analyzed as described in the previous chapter. The reported *PC* is the mean value with standard deviation of all devices analyzed for each liquid. Some channels and devices were excluded from analysis and calculations, e.g.:

- no significant infiltration or very poor infiltration, e.g. stop and go flow
- obvious blockages and optical impairments (bubbles, debris, dust, hair, etc.)
- fits with poor coefficient of determination (< 0.90)
- datasets with very low numbers of interpretable frames (< 10)

Possible reasons for disturbances: incomplete bonding, damages to functionalization surface, procedural or environmental errors, contaminants. Disturbances were more likely to be observed with the *PDMS* devices than with the PMMA chips. This points to laboratory conditions and manual manufacturing as main source of error.

PDMS Devices (SI-Table 8): For water, two of seven manufactured devices (~ 13 channels) yielded plausible results for *PC* with standard deviations < 50%. For complex coacervates, four of six devices (~ 25 channels) with standard deviations < 75% were used for determination of the observed *PC*. The *PC* of ethanol is based on 56/56 channels with standard deviations per chip < 25%, or even < 7%. For iso-propanol, 52/56 channels were successful with standard deviations all < 7%. All nanolime infiltrations were successful with standard deviations < 6%, with two of two successfully infiltrated devices (28/28 channels each).

PMMA Devices (SI-Table 9): For water, 7/8 channels total were infiltrated successfully. For complex coacervates, 7/16 channels total were used for determination of the observed *PC*. Failure was mostly due to total non-infiltration of the liquid. The *PC* of ethanol is based on 6/8 channels. Bubble formation during loading of the chip was the main issue here. For iso-propanol, 4/4 channels were successfully infiltrated. All nanolime infiltrations were successfully performed in 4/4 channels each except NRP-E with 3/4 channels.

Table S5. Results for PC_{obs} in PDMS devices derived from the $PC * r$ values in units of cm/s with their respective standard deviations σ.

	d vs t		d² vs t		d vs t$^{1/2}$	
**Liquid**	PC_{obs} [cm/s]	σ [cm/s]	PC_{obs} [cm/s]	σ [cm/s]	PC_{obs} [cm/s]	σ [cm/s]
**Water**	1226	239	910	72	1255	211
**CC**	1130	376	645	185	1428	300
**EtOH**	845	76	680	56	846	77
**NRP-E**	888	71	723	50	885	68
**CLS-E**	843	45	712	39	843	45
**iPrOH**	427	28	378	23	427	28
**NRP-iP**	485	26	425	23	486	26
**CLS-iP**	466	25	407	22	466	25
**CLS-iP**	227	3	226	11	227	3

Table S6. Results for PC_{obs} in PMMA devices derived from the $PC * r$ values in units of cm/s with their respective standard deviations σ.

	d vs t		d² vs t		d vs t$^{1/2}$	
**Liquid**	PC_{obs} [cm/s]	σ [cm/s]	PC_{obs} [cm/s]	σ [cm/s]	PC_{obs} [cm/s]	σ [cm/s]
**Water**	731	398	416	82	731	398
**CC**	528	251	404	155	541	244
**EtOH**	696	99	748	79	697	98
**NRP-E**	715	40	734	60	711	40
**CLS-E**	729	64	683	38	749	86
**iPrOH**	387	21	389	24	387	21
**NRP-iP**	461	27	428	25	461	27
**CLS-iP**	402	22	401	29	402	22

III.III. Experimental Infiltration Curves

Exemplary experimental infiltration curve plots are given for *PDMS* and PMMA devices with solvents and complex coacervate (CC) liquids. Nanolime curves are omitted due to their similarity to the solvent curves. All raw microscopy data and extracted meniscus position vs. time data are available on KonDATA[2,3]. An imageJ macro was used for data extraction[4]. Curves were edited for false signals and outliers, e.g. due to bubbles. No curves for no/insufficient infiltration. *PC* from d²-vs-t plots consistently lower than others for *PDMS* and either similar or only slightly deviating in PMMA. Due to this, only d-vs-t and d-vs-t$^{1/2}$ plots will be discussed from here on.

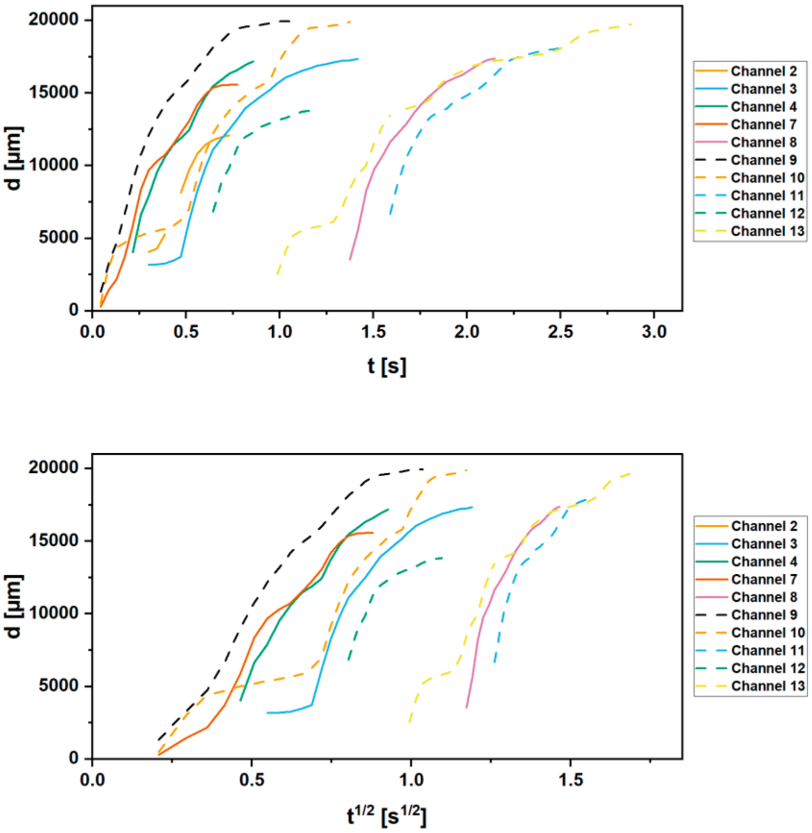

Figure S5. Infiltration of water in PDMS device with 14 observed channels. Top shows d vs t, bottom d vs $t^{1/2}$.

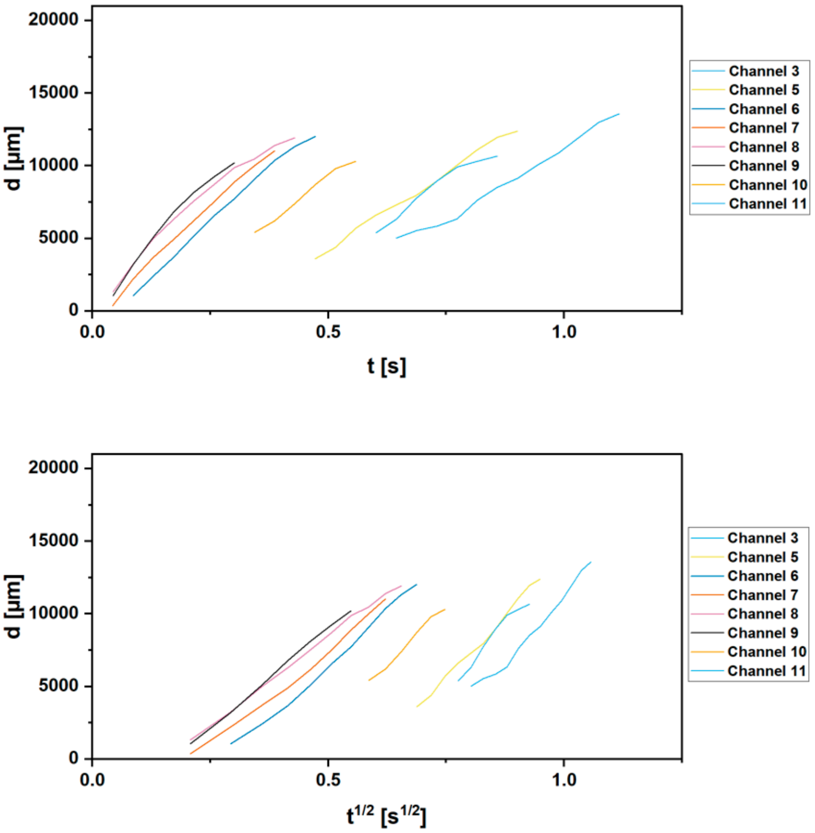

Figure S6. Infiltration data for the complex coacervate formulation in one PDMS device (B) with 14 observed channels. Top shows d vs t, bottom d vs $t^{1/2}$.

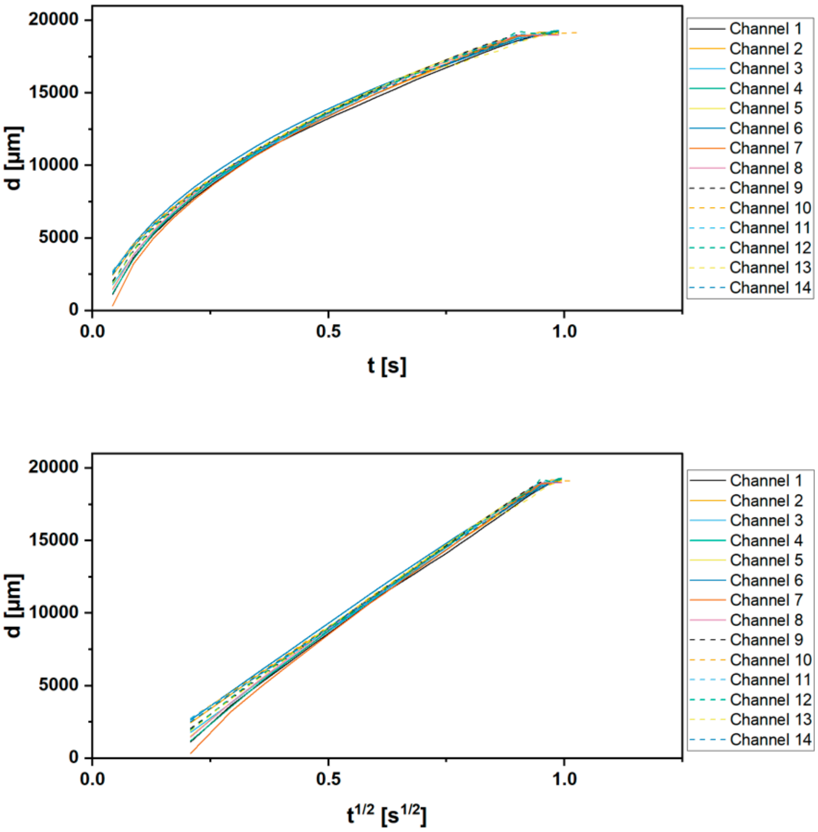

Figure S7. Infiltration data for ethanol in one PDMS device (D) with 14 observed channels. Top shows d vs t, bottom d vs $t^{1/2}$.

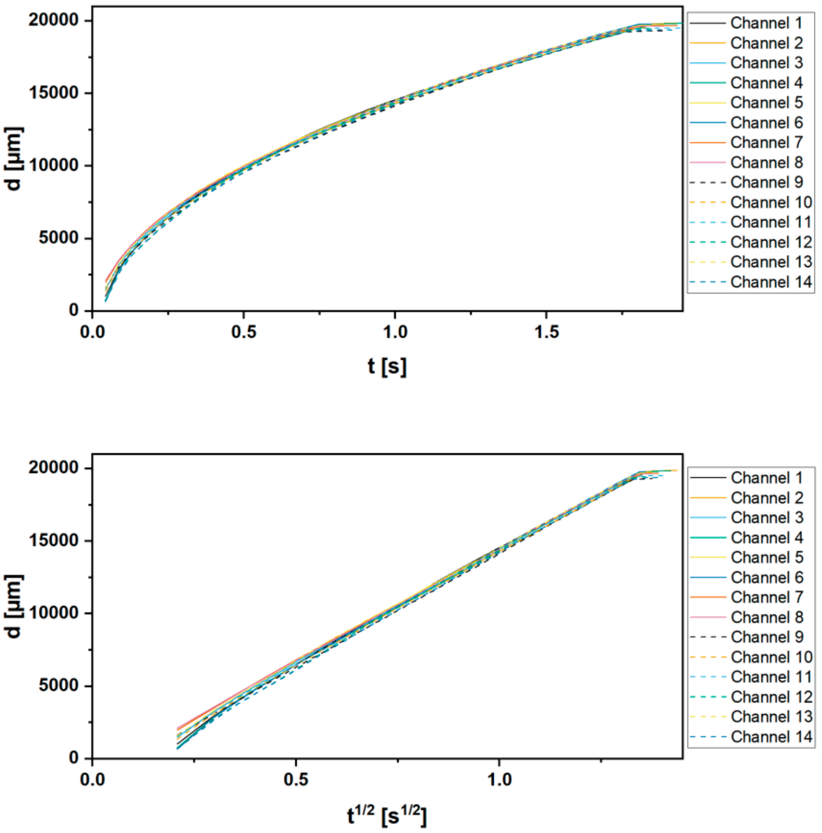

Figure S8. Infiltration data for iso-propanol in one PDMS device (D) with 14 observed channels. Top shows d vs t, bottom d vs $t^{1/2}$.

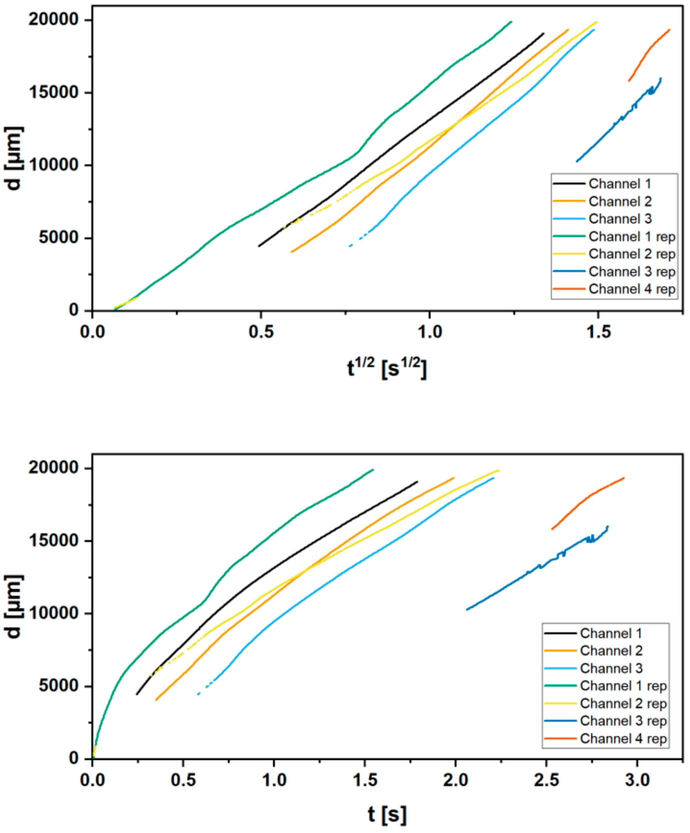

Figure S9. Time-dependent (t) meniscus position (d) plots for water infiltration into PMMA devices. Top shows d vs t, bottom d vs $t^{1/2}$.

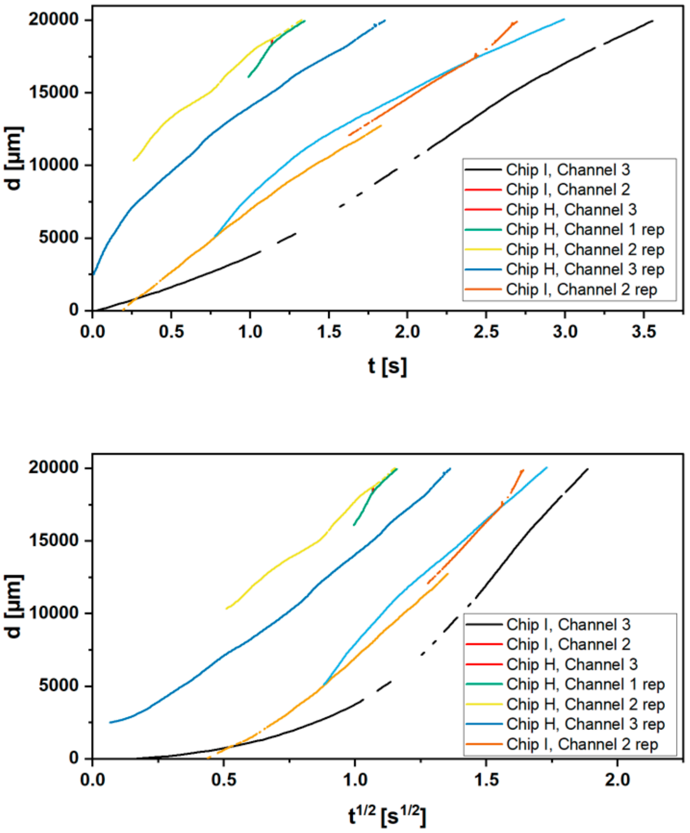

Figure S10. Time-dependent (t) meniscus position (d) plots for complex coacervate (CC) infiltration into PMMA devices. Top shows d vs t, bottom d vs $t^{1/2}$.

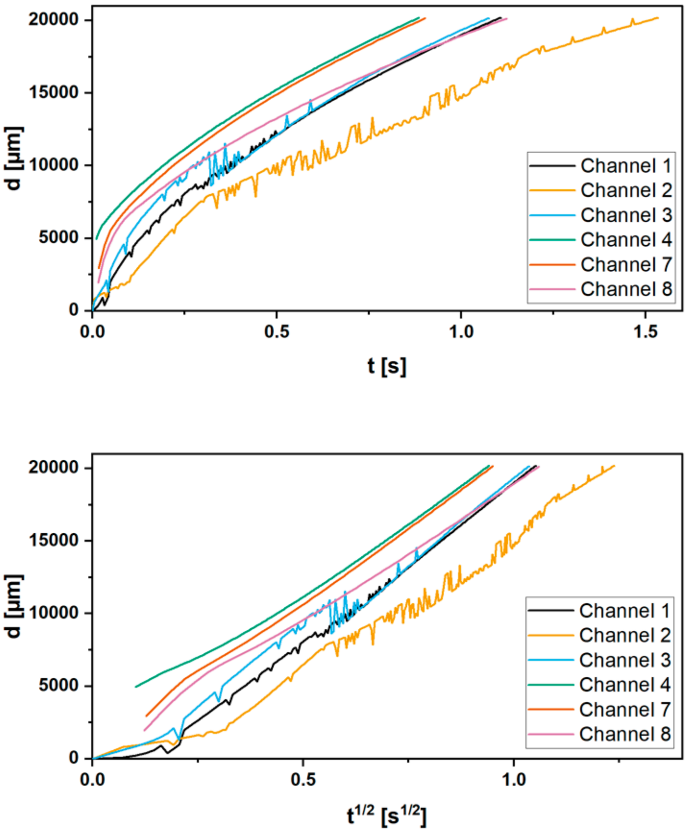

Figure S11. Time-dependent (t) meniscus position (d) plots for ethanol (EtOH) infiltration into PMMA devices. Top shows d vs t, bottom d vs $t^{1/2}$.

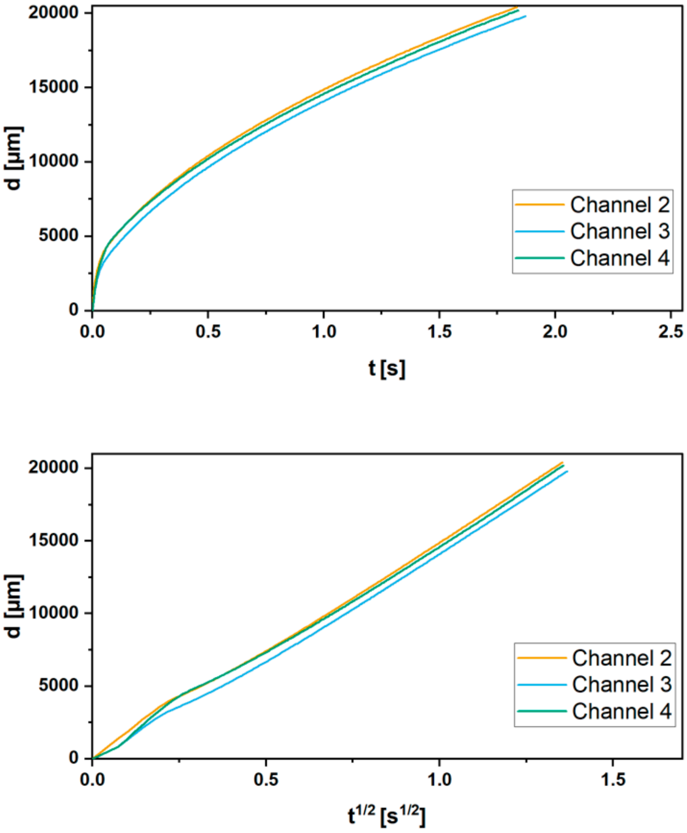

Figure S12. Time-dependent (t) meniscus position (d) plots for iso-propanol (iPrOH) infiltration into PMMA devices. Top shows d vs t, bottom d vs $t^{1/2}$.

III.IV. Glass Resin Micromodel Issues

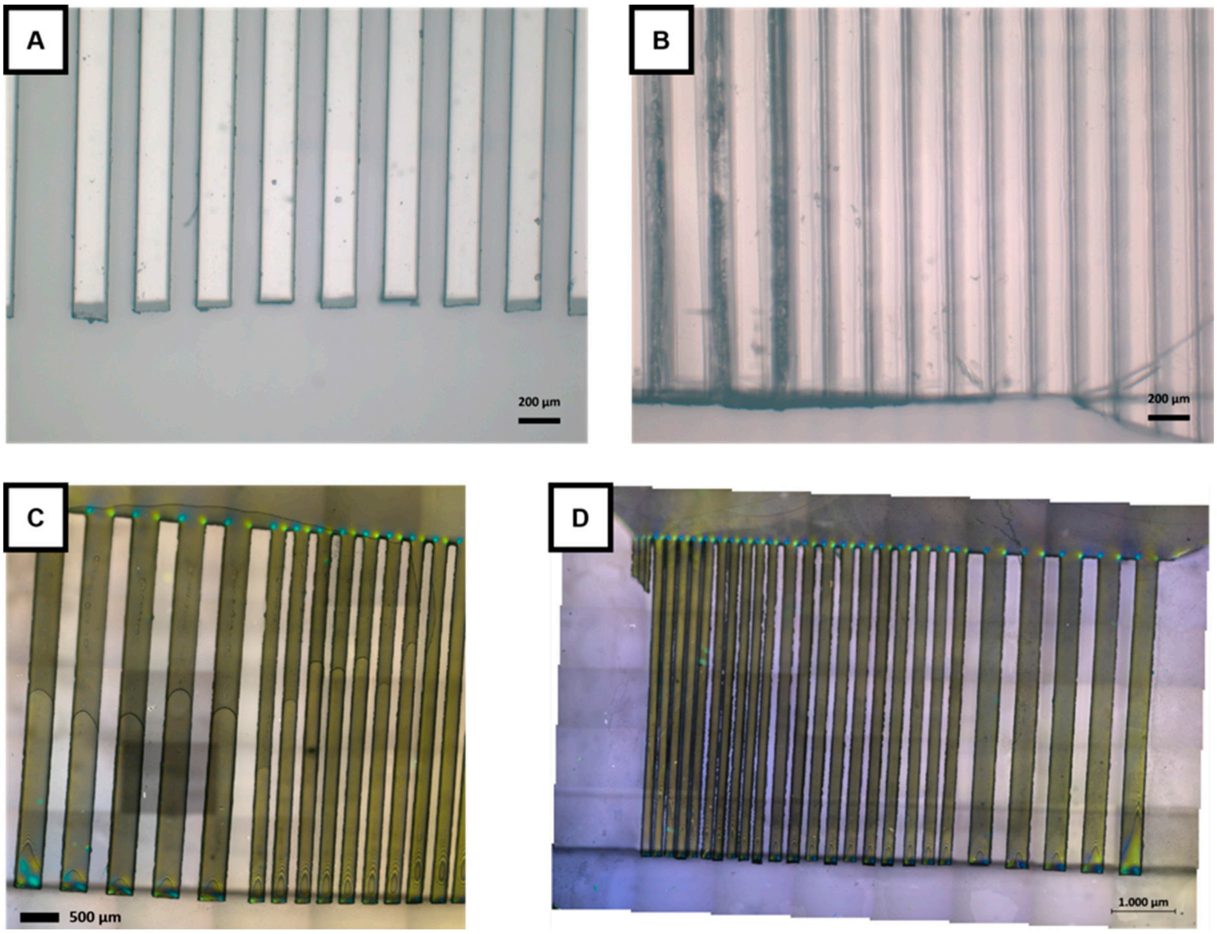

Figure S13. Light microscopy images of SU8 resin-based test systems. Top row shows two different resin structures achieved by photolithography of SU8 photo resin on glass under identical conditions on the same day, resulting in (A) one well-resolved comb structure (lighter structures coming in from the top of the image) and (B) completely fused comb teeth (bridged by thin resin layer, left) and warped comb teeth (pushed together, right). C depicts a system during attempted and failed infiltration: all of the infiltrant has spread between the resin and glass components of the system instead of in the pores. In D an image recorded using an older setup that was available and used in earlier studies is shown, consisting of 56 tiles to depict the whole system.

IV. Theoretical Infiltration Studies

IV.I. Relevant Liquid and Material Properties

There were no literature values available for *CAs* of water on either *PDMS* or *SU8* as modified for this study, also not for nanolimes on the used materials. In these cases, the experimentally obtained contact angles were used for both literature-based and predicted *PC*. Data for viscosity, density, contact angles, and surface tension are available on KonDATA [10].

Table S7. Relevant properties of the solvents and remineralization products used in this study. η: viscosity; ϱ: density; σ: surface tension; θ: contact angle; SU8: resin device; PMMA: poly(methyl methacrylate); NRP: Nanorestore Plus®; CLS: CaLoSiL®; EtOH: ethanol; iPrOH: iso-propanol; CC: complex coacervate.

Solvent Properties	η [mPas]		ϱ [g/cm³]		σ [mN/m]		θ [°]		
	Lit.	Exp.	Lit.	Exp.	Lit.	Exp.	PDMS	PMMA	SU8
Water	1.002 [11],a<br>0.890 [11,12],b	1.0005 [a]<br>0.8913 [b]	0.99821 [11],a<br>0.99705 [11,12],b	0.99822 [a]<br>0.99706 [b]	72.75 [13],a<br>72.01 [13],b	72.67<br>a (+-2 °C)	37.09<br>(5.37)<br>a (+-2 °C)	74.62<br>(3.62)<br>a (+-2 °C)	57.15<br>(3.62)<br>a (+-2 °C)
CC	unk.	1.20644 [a]<br>1.10917 [b]	unk.	1.00782 [a]<br>1.00633 [b]	unk.	71.82<br>a (+-2 °C)	41.77<br>(3.73)<br>a (+-2 °C)	73.10<br>(0.85)<br>a (+-2 °C)	74.00<br>(0.97)<br>a (+-2 °C)
EtOH	1.1890 [14],a<br>1.0995 [14],b	1.1973 [a]<br>1.0853 [b]	0,789377 [14],a<br>0.785087 [14],b	0.789377 [a]<br>0.785087 [b]	22.31 [13],a<br>21.82 [13],b	22.38<br>a (+-2 °C)	11.02<br>(1.03)<br>a (+-2 °C)	0 [c]<br>a (+-2 °C)	0 [c]<br>a (+-2 °C)
NRP-EtOH	1.2 [15]	1.34 [a]<br>1.26 [b]	0.79 [15]	0.79520[a]<br>0.79095[b]	unk.	22.57<br>a (+-2 °C)	0 [c]<br>a (+-2 °C)	0 [c]<br>a (+-2 °C)	0 [c]<br>a (+-2 °C)
CLS-EtOH	1-2 [16],a	1.37 [a]<br>1.25 [b]	0.79-0.83 [16]	0.79769[a]<br>0.79368[b]	unk.	22.68<br>a (+-2 °C)	12.02<br>(0.11)<br>a (+-2 °C)	0[c]<br>a (+-2 °C)	0[c]<br>a (+-2 °C)
iPrOH	2.4140 [11],a<br>2.0700 [11],b	2.4137 [a]<br>2.0663 [b]	0.785350 [11],a<br>0.781100 [11],b	0.78511[a]<br>0.78092[b]	21.74 [13],a<br>21.22 [13],b	21.11<br>a (+-2 °C)	12.46<br>(0.79)<br>a (+-2 °C)	5[d]<br>a (+-2 °C)	5[d]<br>a (+-2 °C)
NRP-iPrOH	2.43 [17]	2.49 [a]<br>2.27 [b]	0.785 [17]	0.79162[a]<br>0.78738[b]	unk.	21.63<br>a (+-2 °C)	6.84<br>(0.96)<br>a (+-2 °C)	5[d]<br>a (+-2 °C)	5[d]<br>a (+-2 °C)
CLS-iPrOH	1-2 [18],a	2.42 [a]<br>2.20 [b]	0.79-0.83 [18]	0.79288[a]<br>0.78866[b]	unk.	19.92<br>a (+-2 °C)	11.58<br>(1.47)<br>a (+-2 °C)	5[d]<br>a (+-2 °C)	5[d]<br>a (+-2 °C)

[a] at 20 °C; [b] at 25 °C; [c] total wetting < 10 s; [d] total wetting < 20 s

IV.II. Simulations of Infiltration Curves

SI-Figure 14 depicts the **simulated curves for modified *PDMS* and PMMA** with all available *PC* values of water (top row), EtOH (middle row), and iPrOH (bottom row) with their respective dispersions. The solvent curves (Figure 4, SI-Figure 14 for 25 °C) demonstrate that larger *PC* values translate to steeper curves in the *d*-vs-*t* plot, with H_2O>EtOH>iPrOH in descending order for all devices. The influence of the parameters of liquid and device material on *PC* and curve steepness follows the LW formula: Higher temperatures *T* result in lower viscosity η, which factors into the equation in the denominator, thereby raising *PC* and the curve steepness. The surface tension γ is part of the

numerator, like r and t, so increasing any of these also increases PC and the curve steepness. In the contact angle range from 0-90 °, where all the liquid and device material combinations fall, an increase in θ leads to a decrease in its cosine value translating to a decrease in PC value and steepness, assuming all else is constant.

Water and CC in ***PDMS*** show that observed and predicted curves are quite similar for both liquids. Also, predicted and literature-based curves for water are indistinguishable. In the EtOH plots, some of the observed curves are so similar, that the observed CLS-E and predicted NRP-E curves, for example, cannot be differentiated from the observed EtOH curve by eye. The upper and lower limit of literature value curves for CLS-E enclose the remaining cluster of similar curves.

In the **PMMA** devices, the trends are mostly the same as in *PDMS* with some exceptions: in the water plot, the predicted CC curve presents steeper than the predicted water curve. Also, the literature and the predicted curves for water are further apart and do not overlap. Overall, water and CC curves in PMMA are less steep than in *PDMS*, while the curves of the other solvents and dispersions do not differ as much in steepness. For the EtOH curves, the observed curves are clustered closely together but further apart from the predicted and literature based EtOH curves than in *PDMS*. For iPrOH plots in all three device materials, all curves except the upper literature value limit can be considered very similar to each other.

Simulations in modified SU8 (*) (SI-Figure 15, left column) for all alcoholic solvents and dispersions are identical to PMMA due to the matching contact angles. The CC simulation for *SU8* only deviates slightly from that of PMMA (ΔPC = 45 cm/s), and the simulated water curve lies between PMMA and *PDMS* water curves with a PC value of 1970 cm/s. The right column of Figure 5 shows PC values and resulting simulated infiltration curves for the modified and native device materials as well as the natural model system, Calcite. Here, we can observe mostly the same trends as before: Water curves are steeper than EtOH curves are steeper than iPrOH curves. Water systems are influenced more by changes in material than alcohols. This is also reflected in contact angles of water and alcohols on these materials, respectively the *PCs* calculated from these. The curves cluster more closely together for EtOH and even closer for iPrOH.

SI-Figure 16 shows exemplary simulated infiltration curves based on values recorded at 25 °C.

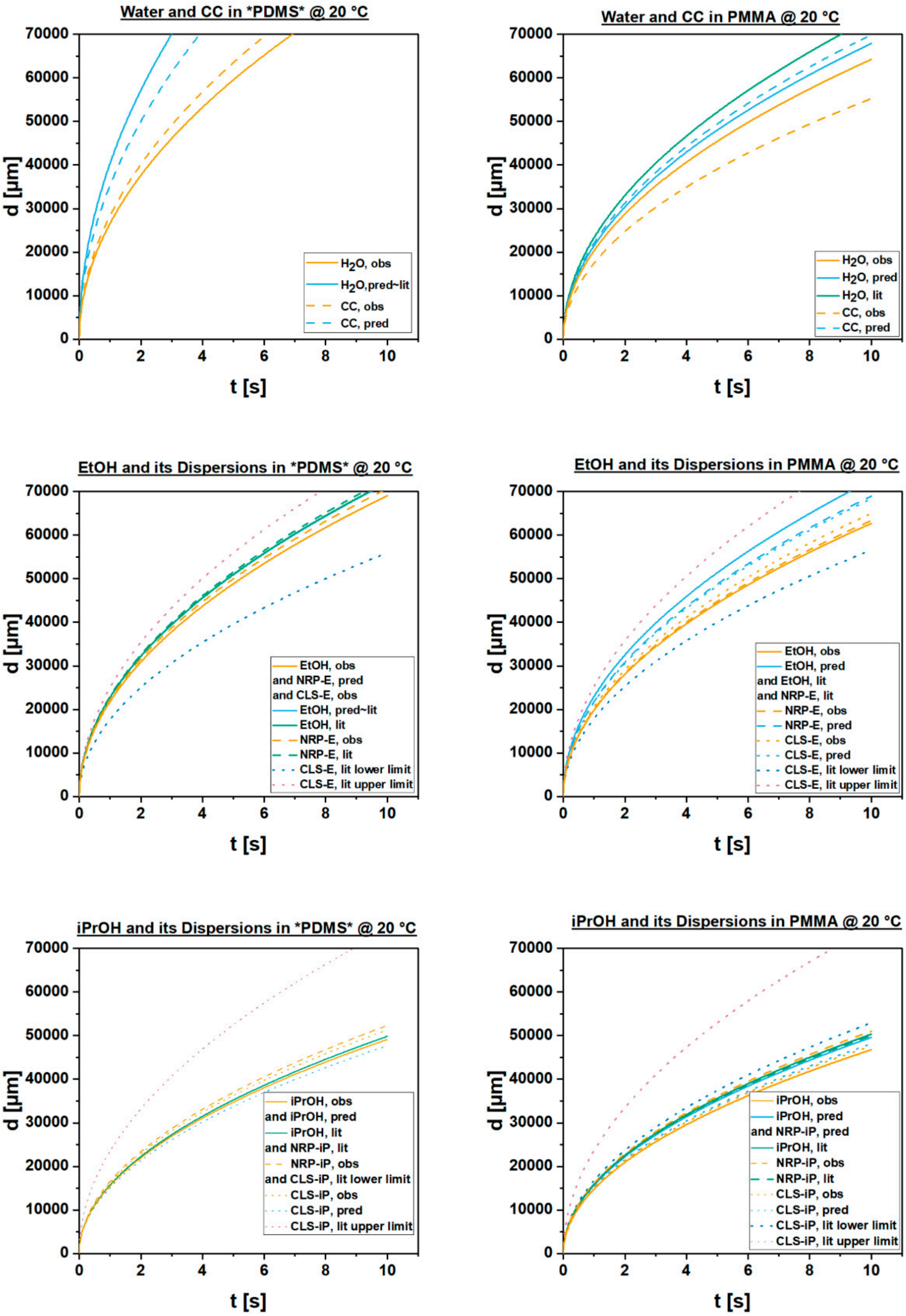

Figure S14. Simulated curves from all *PC* values (obs=observed, pred=predicted and lit=literature-based) for infiltrations into PDMS devices (left column of diagrams) and PMMA devices (right column of diagrams) of different solvents as solid lines (water: top row; EtOH: middle row; iPrOH: bottom row) and respective dispersions (dotted and dashed lines).

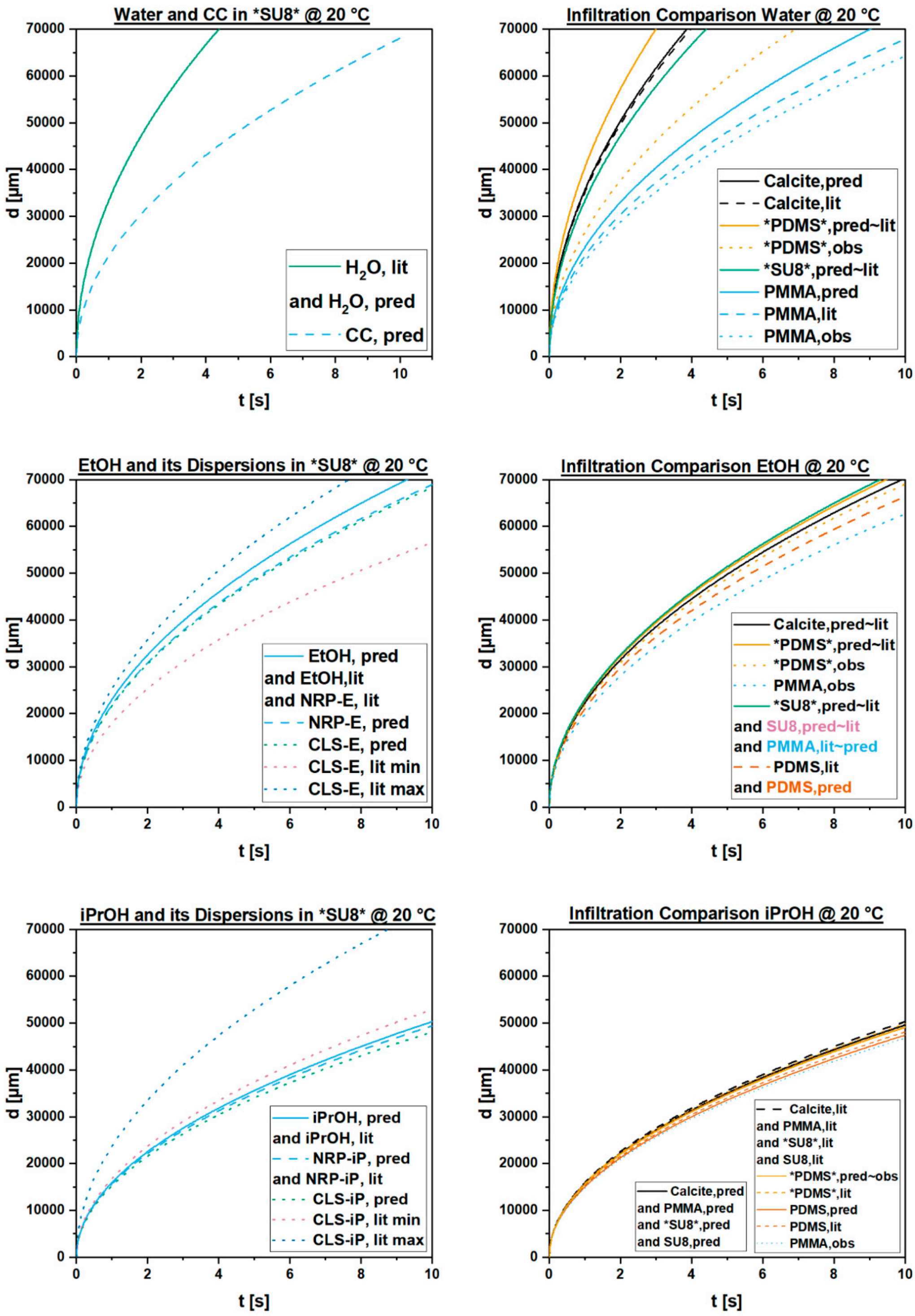

Figure S15. Simulated curves from all *PC* values (obs=observed, pred=predicted and lit=literature-based) for infiltrations of solvents (solid line) and respective dispersions (dotted and dashed lines) into modified *SU8* devices (left column of diagrams). Right column of diagrams show simulated curves for different solvents in device and reference materials. Water: top row; EtOH: middle row; iPrOH: bottom row.

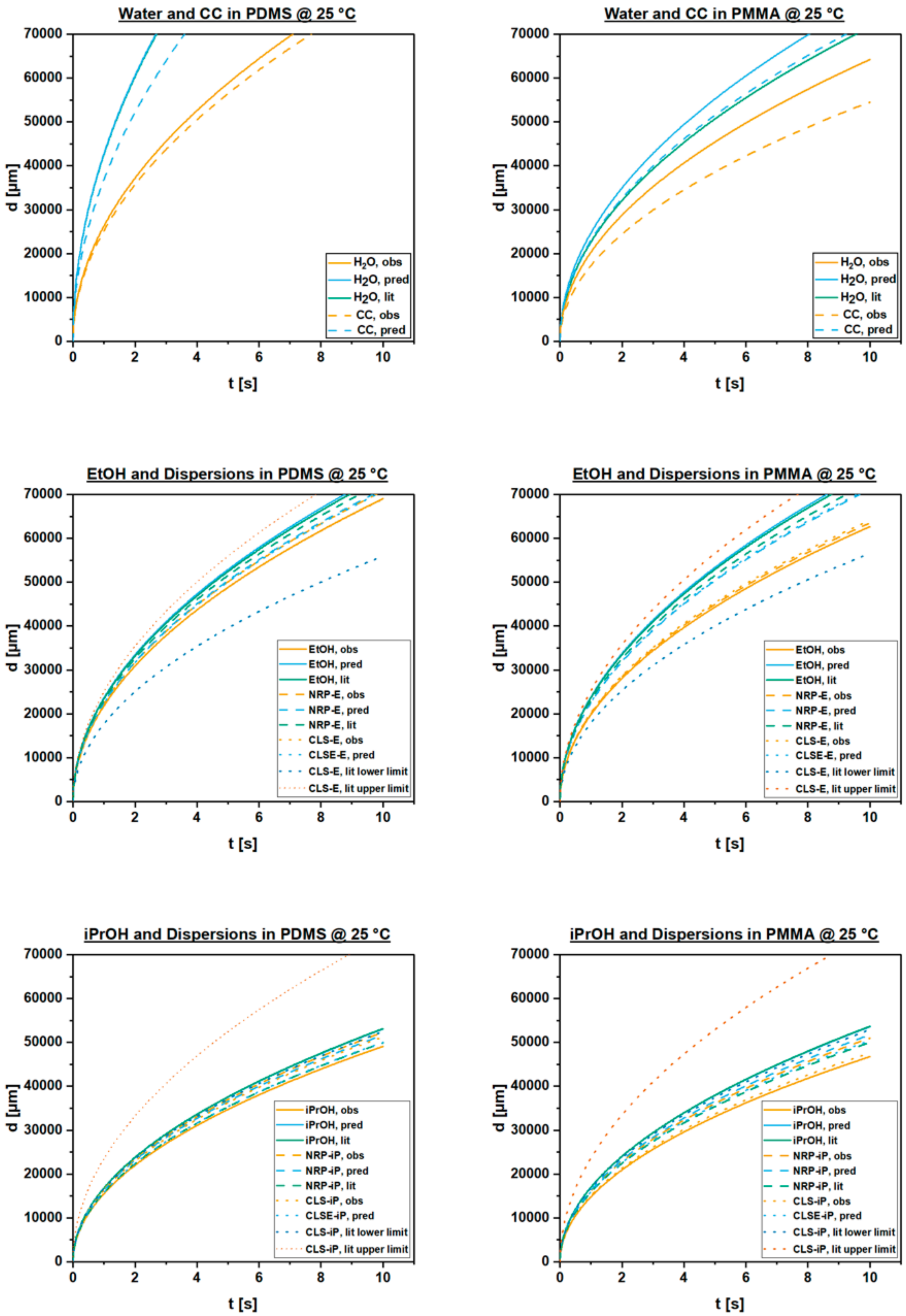

Figure S16. Simulated curves from all *PC* values (observed, predicted, and literature-based) for infiltrations into PDMS (left column of diagrams) and PMMA (right column of diagrams) of different solvents as solid lines (water: top row; EtOH: middle row; iPrOH: bottom row) and respective dispersions (dotted and dashed lines) at 25 °C.Results for Penetration Coefficients.

Table S8. Penetration Coefficients *PC* (with standard deviation) observed during infiltration studies at room temperature (obs), calculated from literature values (lit) and calculated from experimentally determined values (pred) for different combinations of liquids and materials. *SU8*: hard photolithography model, modified; *PDMS*: soft photolithography model, modified; PMMA: commercially available PMMA microfluidic chips, unmodified; CC: complex coacervate of calcium and PAA in water; NRP: Nanorestore Plus®; CLS: CaLoSiL®; EtOH: ethanol; iPrOH: iso-propanol; unk.: unknown.

[cm/s]	*PDMS*			PMMA			*SU8*		
	PC_{obs}	PC_{pred}	PC_{lit}	PC_{obs}	PC_{pred}	PC_{lit}	PC_{obs}	PC_{pred}	PC_{lit}
Water	1255 (212)	2897 [a]<br>3252 [b]	2896 [a]<br>3227 [b]	731 (398)	963 [a]<br>1081 [b]	817 [a]<br>910 [b]	-	1970 [a]<br>2211 [b]	1969 [a]<br>2194 [b]
CC in Water	1428 (300)	2220 [a]<br>2415 [b]	unk.	541<br>(244)	865 [a]<br>941 [b]	unk.	-	820 [a]<br>892 [b]	unk.
EtOH	846 (77)	917 [a]<br>991 [b]	921 [a]<br>974 [b]	697 (98)	935 [a]<br>1009 [b]	938 [a]<br>992 [b]	-	935 [a]<br>1009 [b]	938 [a]<br>992 [b]
NRP-EtOH	885 (68)	842 [a]<br>898 [b]	940 [c]	711 (40)	842 [a]<br>898 [b]	940 [c]	-	842 [a]<br>898 [b]	940 [c]
CLS-EtOH	843 (45)	810 [a]<br>887 [b]	555 –1109 [c,d]	749<br>(86)	828 [a]<br>907 [b]	567 –1134 [c,d]	-	828 [a]<br>907 [b]	567 –1134 [c,d]
iPrOH	427 (28)	427 [a]<br>499 [b]	440 [a]<br>500 [b]	387 (21)	436 [a]<br>509 [b]	449 [a]<br>511 [b]	-	436 [a]<br>509 [b]	449 [a]<br>511 [b]
NRP-iPrOH	486 (26)	431 [a]<br>473 [b]	442 [c]	461 (27)	433 [a]<br>475 [b]	443 [c]	-	433 [a]<br>475 [b]	443 [c]
CLS-iPrOH	466 (25)	403 [a]<br>444 [b]	488 – 976 [c,d]	402 (22)	410 [a]<br>451 [b]	496 –992 [c,d]	-	410 [a]<br>451 [b]	496 –992 [c,d]

[a] at 20 °C; [b] at 25 °C; [c] temperature not specified; [d] upper and lower limit of reported viscosity

Table S9. Penetration Coefficients *PC* (with standard deviation) calculated from literature (lit) and from experimentally determined values (pred) for different liquid/reference material combinations. CC: complex Ca/PAA coacervate in water; NRP: Nanorestore Plus®; CLS: CaLoSiL®; EtOH: ethanol; iPrOH: iso-propanol.

[cm/s]	Calcite		Native PDMS		Native SU8	
	PC_{pred}	PC_{lit}	PC_{pred}	PC_{lit}	PC_{pred}	PC_{lit}
Water	2244[a]<br>2519[b]	2185[a]<br>2435[b]	-1423[a]<br>-1597[b]	-1422[a]<br>-1585[b]	-119[a]<br>-133[b]	-118[a]<br>-132[b]
CC in Water	1964[a]<br>2136[b]	-	-1199[a]<br>-1304[b]	-	-97[a]<br>-106[b]	-
EtOH	876 [a]<br>947[b]	880[a]<br>931[b]	778[a]<br>840[b]	781[a]<br>826[b]	926[a]<br>1000[b]	930[a]<br>983[b]
NRP-EtOH	786[a]<br>838[b]	878[c]	701[a]<br>748[b]	783[c]	838[a]<br>894[b]	936[c]
CLS-EtOH	777[a]<br>852[b]	532 –<br>1065[d]	705[a]<br>773[b]	483 –<br>966[d]	823[a]<br>802[b]	564 –<br>1128[d]
iPrOH	436[a]<br>509[b]	449[a]<br>511[b]	398[a]<br>465[b]	410[a]<br>567[b]	437[a]<br>511[b]	450[a]<br>513[b]
NRP-iPrOH	433[a]<br>475[b]	443[c]	390[a]<br>428[b]	399[c]	434[a]<br>476[b]	445[c]
CLS-iPrOH	410[a]<br>451[b]	496 –<br>992[d]	378[a]<br>416[b]	457 –<br>915[d]	412[a]<br>453[b]	498 –<br>996[d]

[a] at 20 °C; [b] at 25 °C; [c] temperature not specified; [d] upper and lower limit of reported viscosity

V. Predictions for Calcite

Conversions between *PDMS* and PMMA were executed for identical liquids using

- PC_{obs} values and derived CAs thereof (SI-Table 13)
- PC_{pred} values and experimentally determined CAs (SI-Table 14)
- PC_{obs} values and experimentally determined CAs (SI-Table 15)

Contact angle calculation from PC_{obs}: $\theta_{calc} = \cos^{-1}\frac{PC_{obs}*2\eta}{\sigma}$

Table S10. PC_{obs} (*PC*) values of *PDMS* and PMMA, as well as the cPC_{*PDMS*}(PMMA)of *PDMS* converted from PMMA and the cPC_{PMMA}(*PDMS*) of PMMA converted from *PDMS* (PC_{*PDMS*}) using CAs calculated from PC_{obs} values.

Liquid	PC_{*PDMS*} [cm/s]	cPC_{*PDMS*}(PMMA) [cm/s]	PC_{PMMA} [cm/s]	cPC_{PMMA}(*PDMS*) [cm/s]
Water	1255	846	731	1085
CC	1428	885	541	873
EtOH	846	846	697	697
iPrOH	427	427	387	387

Table S11. PC_{pred} (*PC*) values of *PDMS* and PMMA, as well as the predicted *PCs* of *PDMS* converted from PMMA (PC_{PMMA}) and the *PCs* of PMMA converted from *PDMS* (PC_{*PDMS*}) using experimentally measured CAs.

Liquid	PC_{*PDMS*} [cm/s]	cPC_{*PDMS*}(PMMA) [cm/s]	PC_{PMMA} [cm/s]	cPC_{PMMA}(*PDMS*) [cm/s]
Water	2897	2897	963	963
CC	2220	2220	865	865
EtOH	917	917	935	935
iPrOH	427	390	398	436

Table S12. PC_{obs} (*PC*) values of *PDMS* and PMMA, as well as the predicted *PCs* of *PDMS* converted from PMMA (PC_{PMMA}) and the *PCs* of PMMA converted from *PDMS* (PC_{*PDMS*}) using experimentally measured CAs.

Liquid	PC_{*PDMS*} [cm/s]	cPC_{*PDMS*}(PMMA) [cm/s]	PC_{PMMA} [cm/s]	cPC_{PMMA}(*PDMS*) [cm/s]
Water	1255	2199	731	417
CC	1428	1387	541	557
EtOH	846	684	697	862
iPrOH	427	388	387	436

Conversions between different alcohols and water/CC were executed for identical device materials using:

- PC_{obs} and derived *CAs* thereof for *PDMS* (SI-Table 16) and PMMA (SI-Table 17)
- PC_{pred} and experimental det. *CAs* for *PDMS* (SI-Table 18) and PMMA (SI-Table 19)

Table S13. *cPC* results converted from one liquid to another in *PDMS* devices from PC_{obs} and CAs thereof.

cPC_{*PDMS*} [cm/s] *based on:*	Water	CC	EtOH	iPrOH
Water	-	1312	-	-
CC	1366	-	-	-
EtOH	-	-	-	427

iPrOH	-	-	846	-

Table S14. *cPC* results converted from one liquid to another in PMMA devices from PC_{obs} and CAs thereof.

cPC*$_{PMMA}$** **[cm/s]** ***based on:	**Water**	**CC**	**EtOH**	**iPrOH**
Water	-	541	-	-
CC	731	-	-	-
EtOH	-	-	-	427
iPrOH	-	-	846	-

Table S15. *cPC* results converted from one liquid to another in *PDMS* devices from PC_{pred} and experimentally determined CAs.

cPC*$_{*PDMS*}$** **[cm/s]** ***based on:	**Water**	**CC**	**EtOH**	**iPrOH**
Water	-	2220	-	-
CC	2897	-	-	-
EtOH	-	-	-	427
iPrOH	-	-	917	-

Table S16. *cPC* results converted from one liquid to another in PMMA devices from PC_{pred} and experimentally determined CAs.

cPC*$_{PMMA}$** **[cm/s]** ***based on:	**Water**	**CC**	**EtOH**	**iPrOH**
Water	-	865	-	-
CC	963	-	-	-
EtOH	-	-	-	436
iPrOH	-	-	854	-

VI. Error Calculations

Mean values $\bar{x}$ of variable x from n results x_i : $\bar{x} = \frac{x_1+x_2+\cdots+x_n}{n} = \frac{1}{n}\sum_{i=1}^{n} x_i$

Variance $\sigma_x^2 = \frac{1}{n}\sum_{i=1}^{n}(x_i - \bar{x})^2$ and standard deviation $\sigma_x = \sqrt{\sigma_x^2}$

For PC_{obs} (*F*), the Gaussian Error Propagation was employed using variables $B=PC^*r$ from curve fitting and radius r with their respective errors ΔB and $\Delta r \sim 3$ µm:

$$\Delta(F) = \sqrt{\left(\frac{\partial F}{\partial x} * \Delta(x)\right)^2 + \left(\frac{\partial F}{\partial y} * \Delta(y)\right)^2 + \left(\frac{\partial F}{\partial z} * \Delta(z)\right)^2}$$

References

1. Schindelin, J.; Arganda-Carreras, I.; Frise, E.; Kaynig, V.; Longair, M.; Pietzsch, T.; Preibisch, S.; Rueden, C.; Saalfeld, S.; Schmid, B.; et al. Fiji: an open-source platform for biological-image analysis. *Nat Methods* **2012**, *9*, 676–682, doi:10.1038/nmeth.2019.
2. Infiltration into PDMS - Light Microscopy Data. Available online: https://kondata.uni-konstanz.de/radar/de/dataset/VktwYLYPSiLvOZFO.Infiltration%2BPDMS%2B-%2BLight%2BMicroscopy%2BData (accessed on 31 January 2023).

3. Infiltration into PMMA - Light Microscopy Data. Available online: https://kondata.uni-konstanz.de/radar/de/dataset/LidnocgGQVeXGfUe.Infiltration%2BPMMA%2B-%2BLight%2BMicroscopy%2BData (accessed on 31 January 2023).
4. Stöckl, M.T. *Liquid Meniscus Tracking ImageJ Macro*. Image J Makro; Zenodo: Konstanz, 2022.
5. Hoyt, A.-L.M.; Cölfen, H. Stone pore imitating artificial test systems : Current prototypes and potential for cultural heritage care. In *Monument Future*, Decay and Conservation of Stone, 14th International Congress on the Deteriotation and Conservation of Stone; Siegesmund, S., Middendorf, B., Eds.; Mitteldeutscher Verlag: Halle, 2020; pp 573–578, ISBN 978-3-96311-172-3.
6. Gruber, D.; Wolf, S.L.P.; Hoyt, A.-L.M.; Konsek, J.P.; Cölfen, H. A Micro-Comb Test System for In Situ Investigation of Infiltration and Crystallization Processes. *Minerals* **2017**, *7*, 187, doi:10.3390/min7100187.
7. Erfani Gahrooei, H.R.; Ghazanfari, M.H. Toward a hydrocarbon-based chemical for wettability alteration of reservoir rocks to gas wetting condition: Implications to gas condensate reservoirs. *Journal of Molecular Liquids* **2017**, *248*, 100–111, doi:10.1016/j.molliq.2017.10.043.
8. Demming, S.; Lesche, C.; Schmolke, H.; Klages, C.-P.; Büttgenbach, S. Characterization of long-term stability of hydrophilized PEG-grafted PDMS within different media for biotechnological and pharmaceutical applications. *Physica status solidi (a)* **2011**, *208*, 1301–1307.
9. Jokinen, V.; Suvanto, P.; Franssila, S. Oxygen and nitrogen plasma hydrophilization and hydrophobic recovery of polymers. *Biomicrofluidics* **2012**, *6*, 16501.
10. Experimental Properties of Solvents, Nanolimes, and a Complex Coacervate. Available online: https://kondata.uni-konstanz.de/radar/de/dataset/AiQGnvCAnPOiSEoJ.Experimental%2BProperties%2BSolvents%252C%2BNanolimes%252C%2BComplex%2BCoacervate (accessed on 31 January 2023).
11. Pang, F.-M.; Seng, C.-E.; Teng, T.-T.; Ibrahim, M.H. Densities and viscosities of aqueous solutions of 1-propanol and 2-propanol at temperatures from 293.15 K to 333.15 K. *Journal of Molecular Liquids* **2007**, *136*, 71–78, doi:10.1016/j.molliq.2007.01.003.
12. Gonzalez, B.; Dominguez, A.; Tojo, J. Dynamic viscosities, densities, and speed of sound and derived properties of the binary systems acetic acid with water, methanol, ethanol, ethyl acetate and methyl acetate at T = (293.15, 298.15, and 303.15) K at atmospheric pressure. *J Chem Eng Data* **2004**, *49*, 1590–1596, doi:10.1021/je0342825.
13. Vazquez, G.; Alvarez, E.; Navaza, J.M. Surface-Tension of Alcohol Plus Water from 20-Degrees-C to 50-Degrees-C. *J Chem Eng Data* **1995**, *40*, 611–614, doi:10.1021/je00019a016.
14. Khattab, I.S.; Bandarkar, F.; Fakhree, M.A.A.; Jouyban, A. Density, viscosity, and surface tension of water+ ethanol mixtures from 293 to 323K. *Korean Journal of Chemical Engineering* **2012**, *29*, 812–817.
15. SDS. *Safety Data Sheet Nanorestore Plus(R) 5 g/L in Ethanol*, Florence (Italy), 2016.
16. SDS. *Safety Data Sheet CaLoSiL(R) E-Products up until E25*, Halsbrücke (Germany), 2017.
17. SDS. *Safety Data Sheet Nanorestore Plus(R) 5 g/L in iso-Propanol*, Florence (Italy), 2016.
18. SDS. *Safety Data Sheet CaLoSiL(R) IP-Products, iso-Propanol-based*, Halsbrücke (Germany), 2017.
